# Supplementary material for: Performance of deep learning to detect mastoiditis using multiple conventional radiographs of mastoid
Source: PLoS One. 2020 Nov 11;15(11):e0241796. doi: 10.1371/journal.pone.0241796 (PMC7657495; doi:10.1371/journal.pone.0241796)

## Slide 1
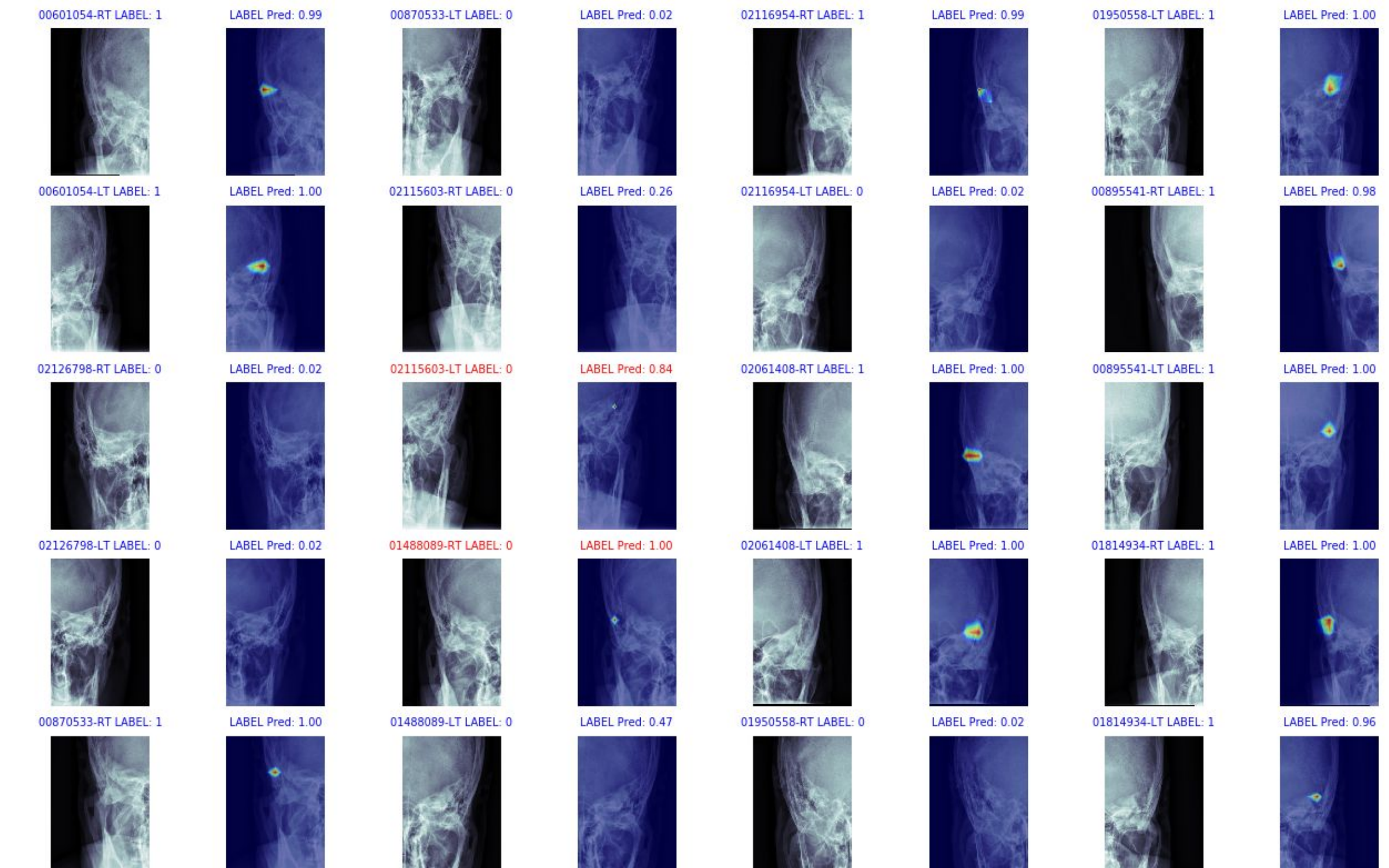

## Slide 2
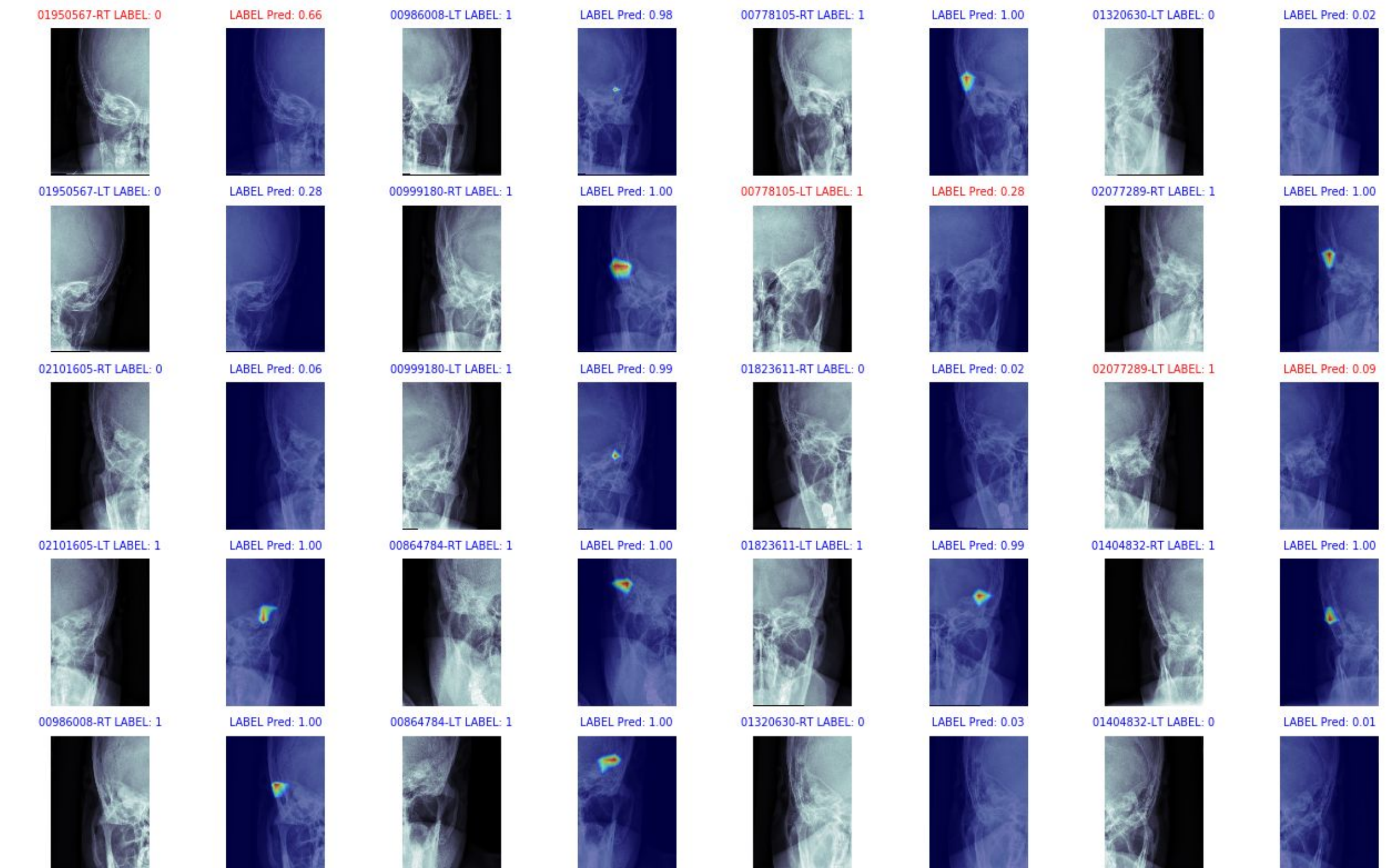

## Slide 3
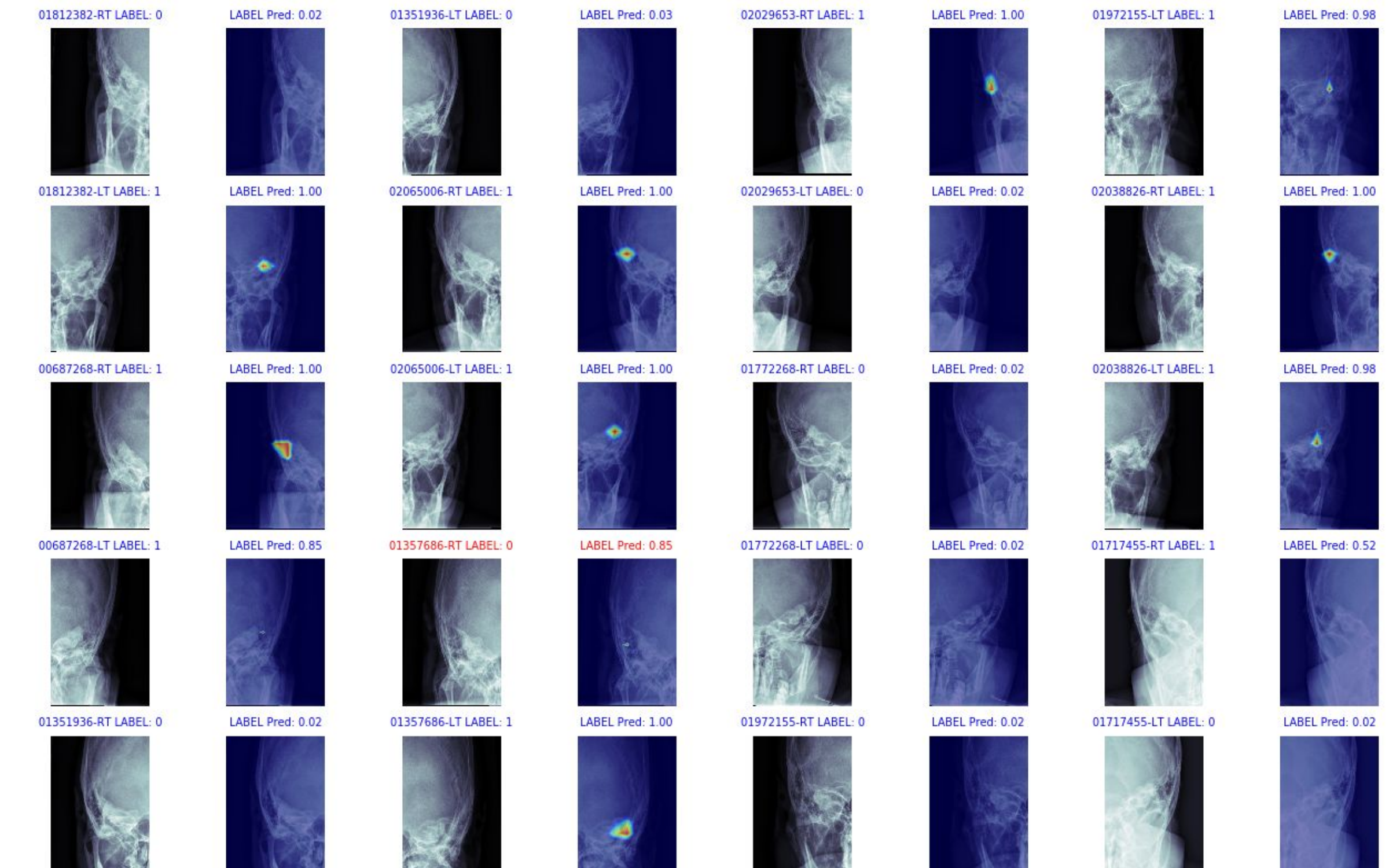

## Slide 4
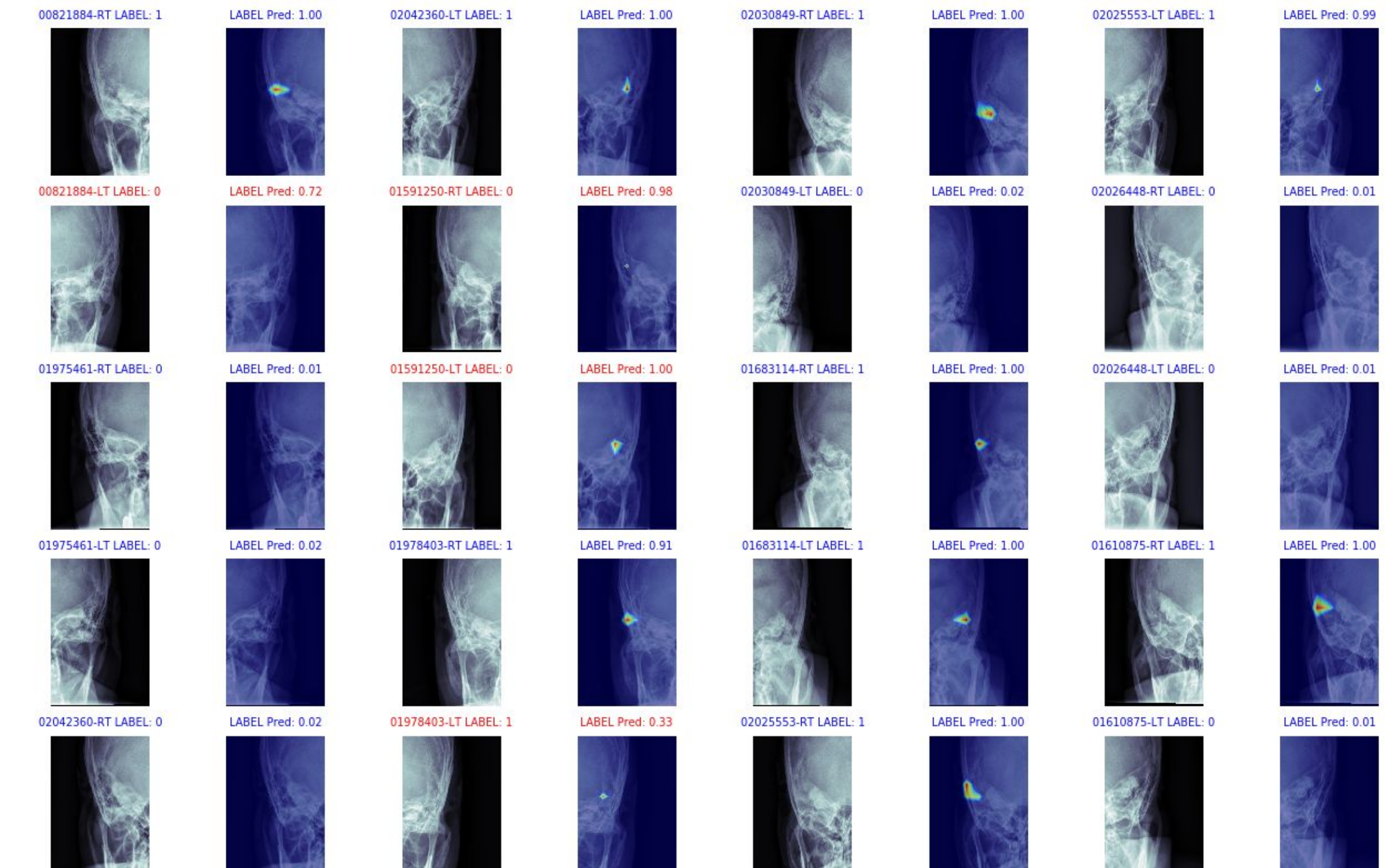

## Slide 5
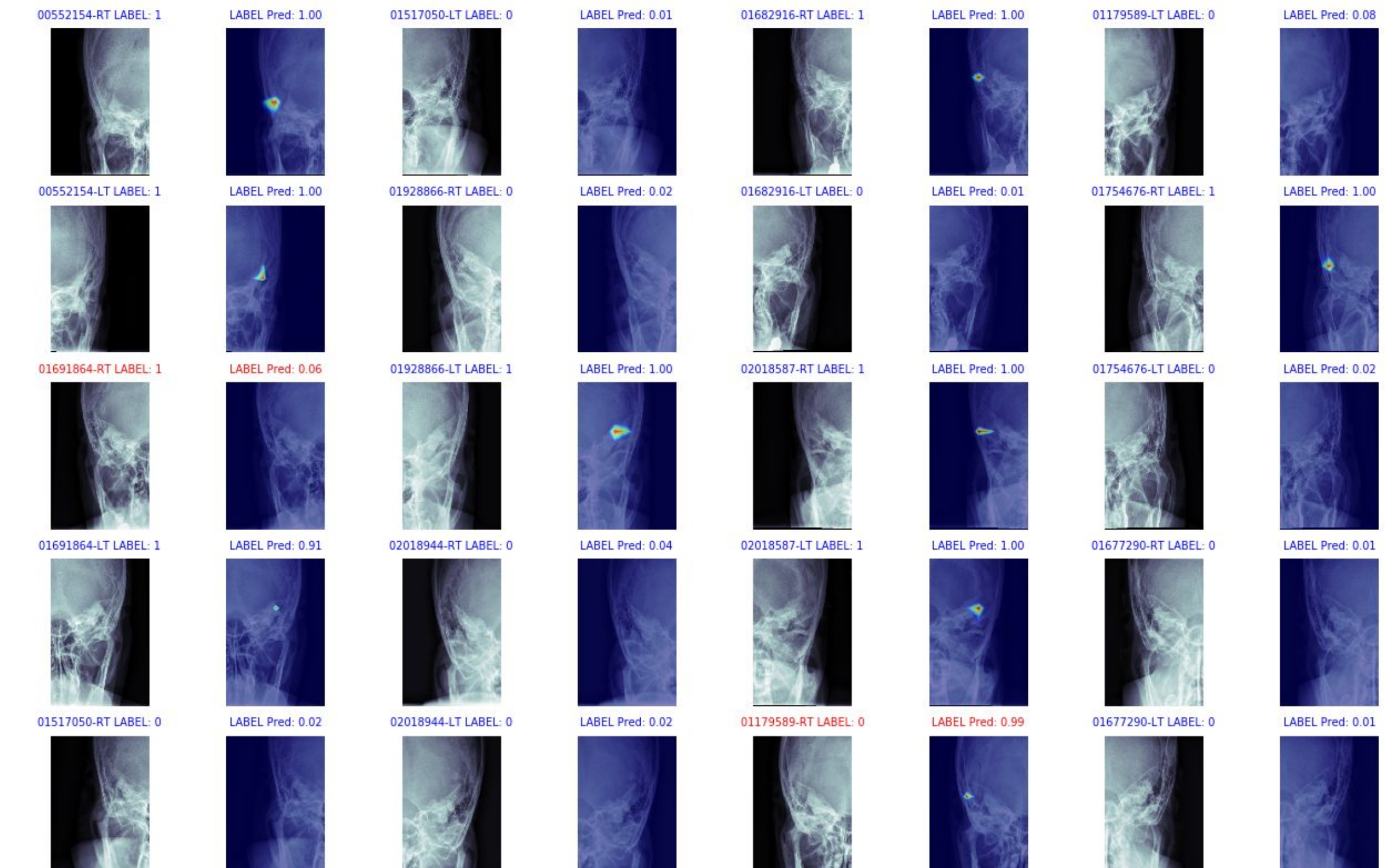

## Slide 6
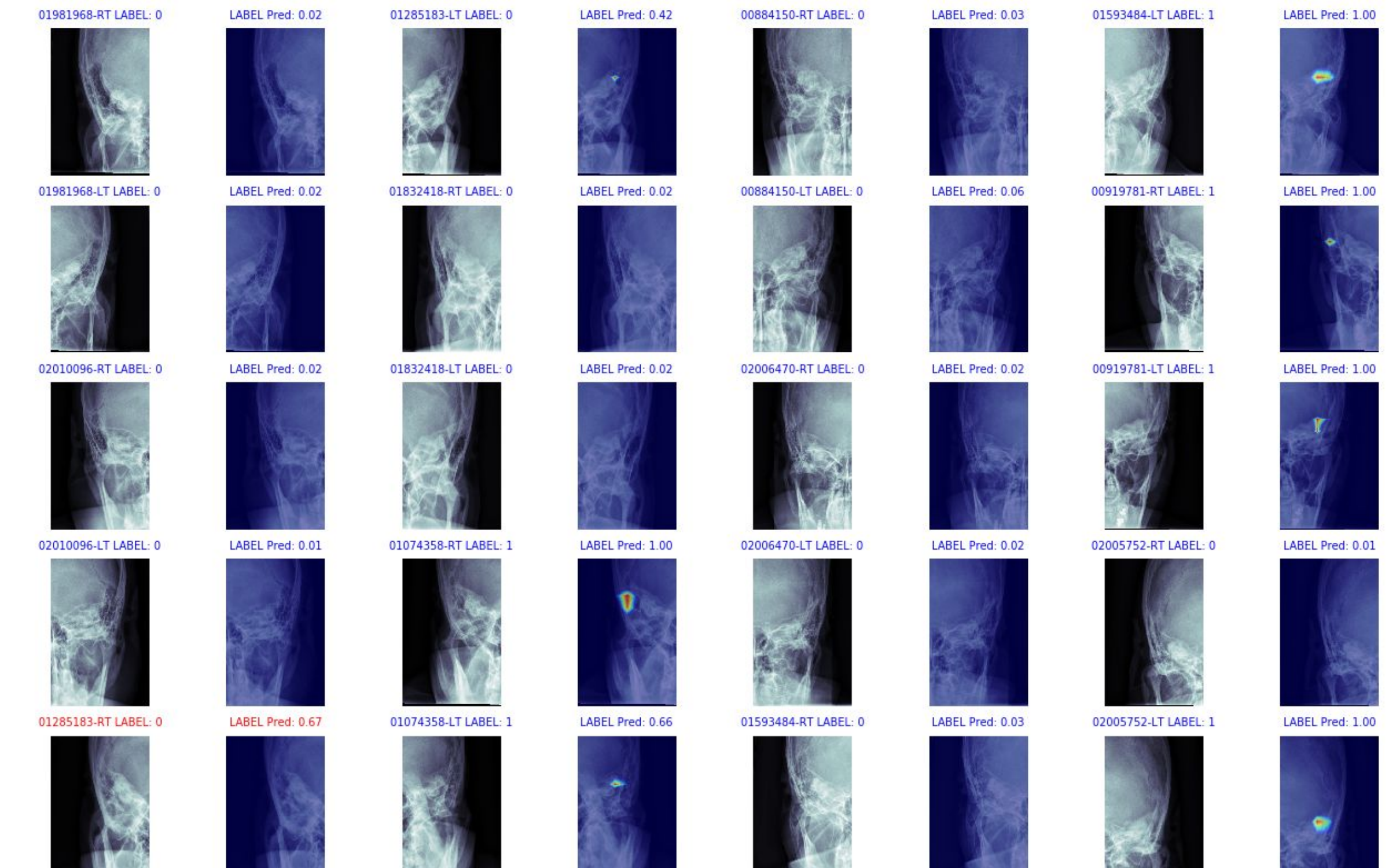

## Slide 7
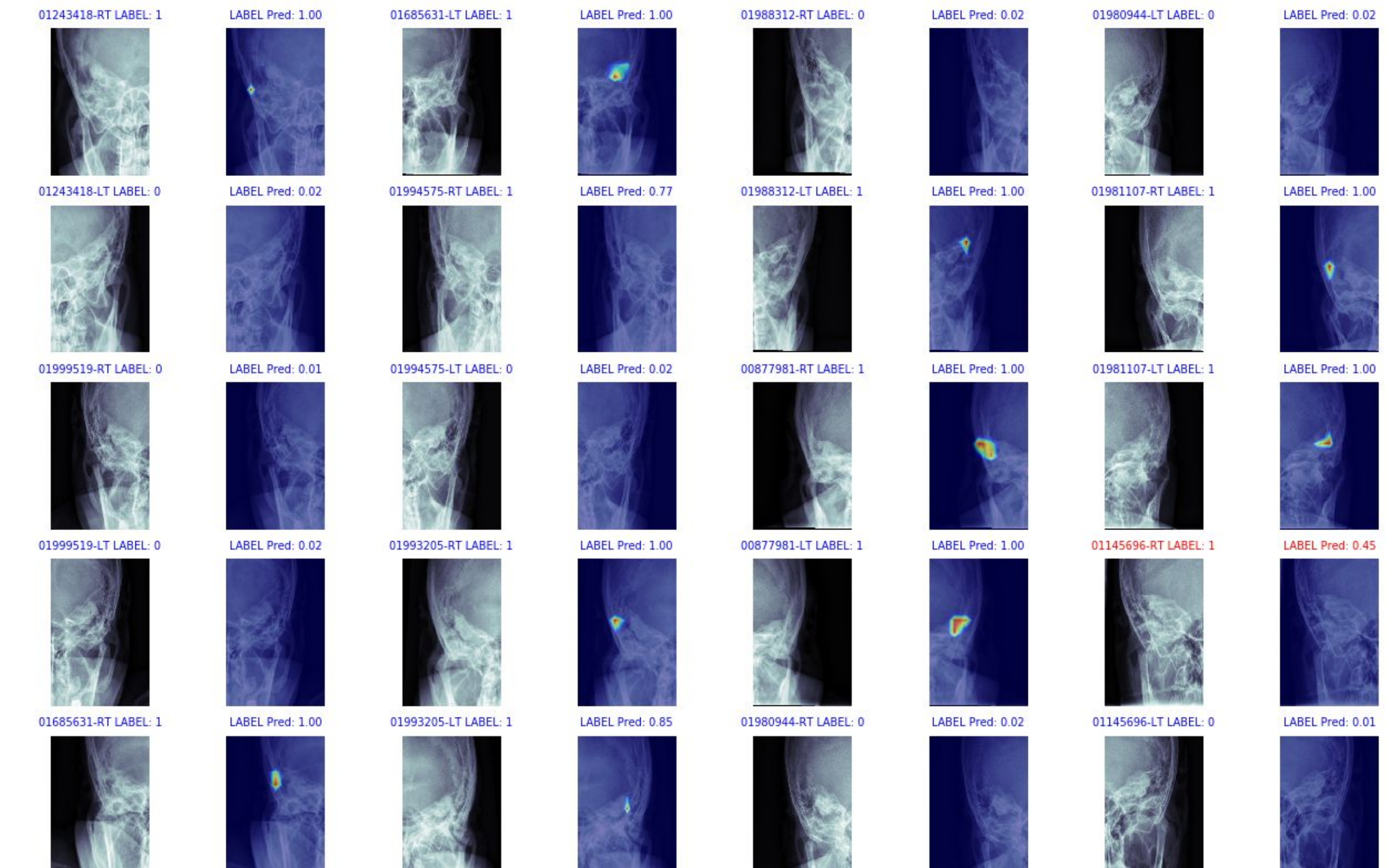

## Slide 8
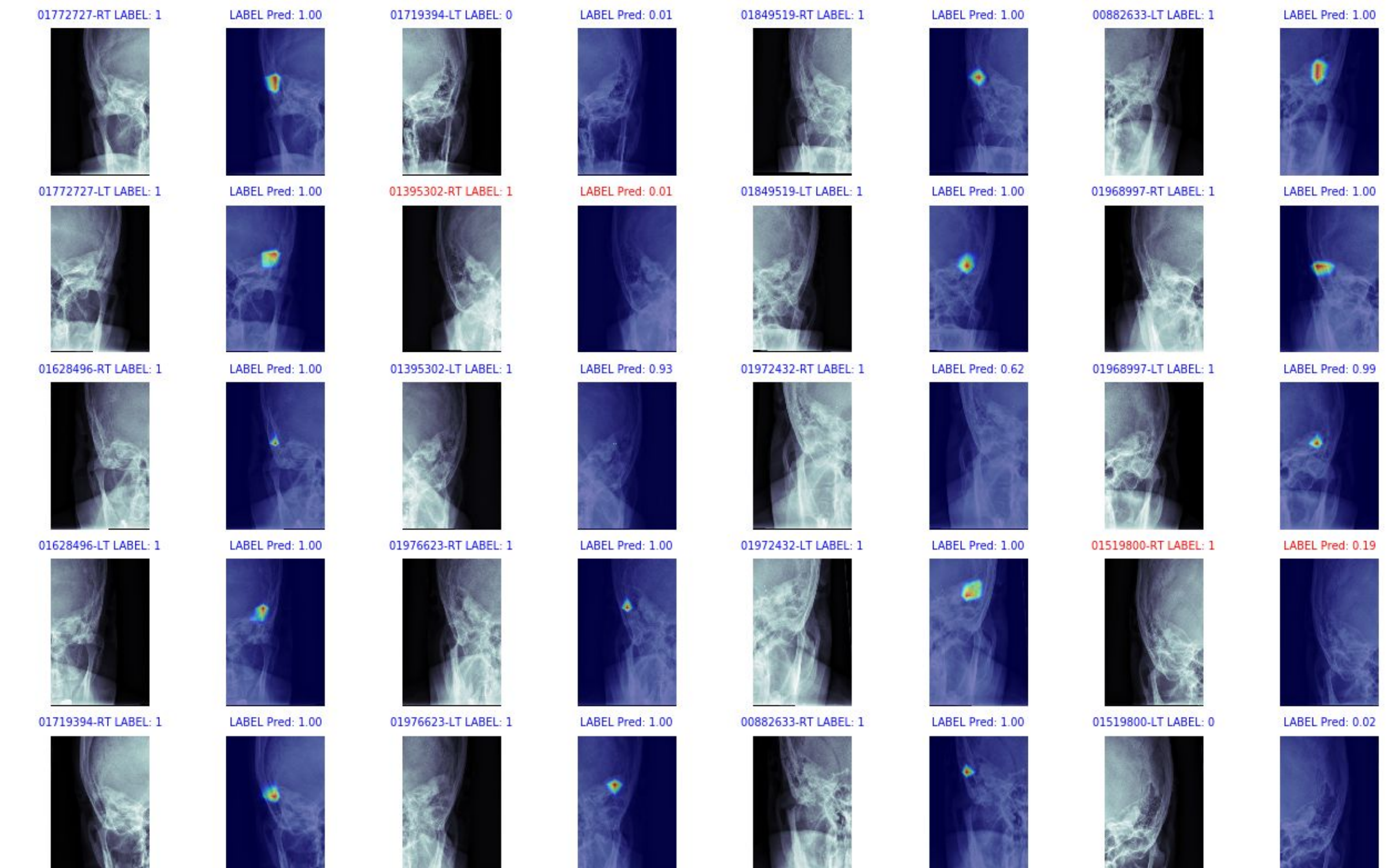

## Slide 9
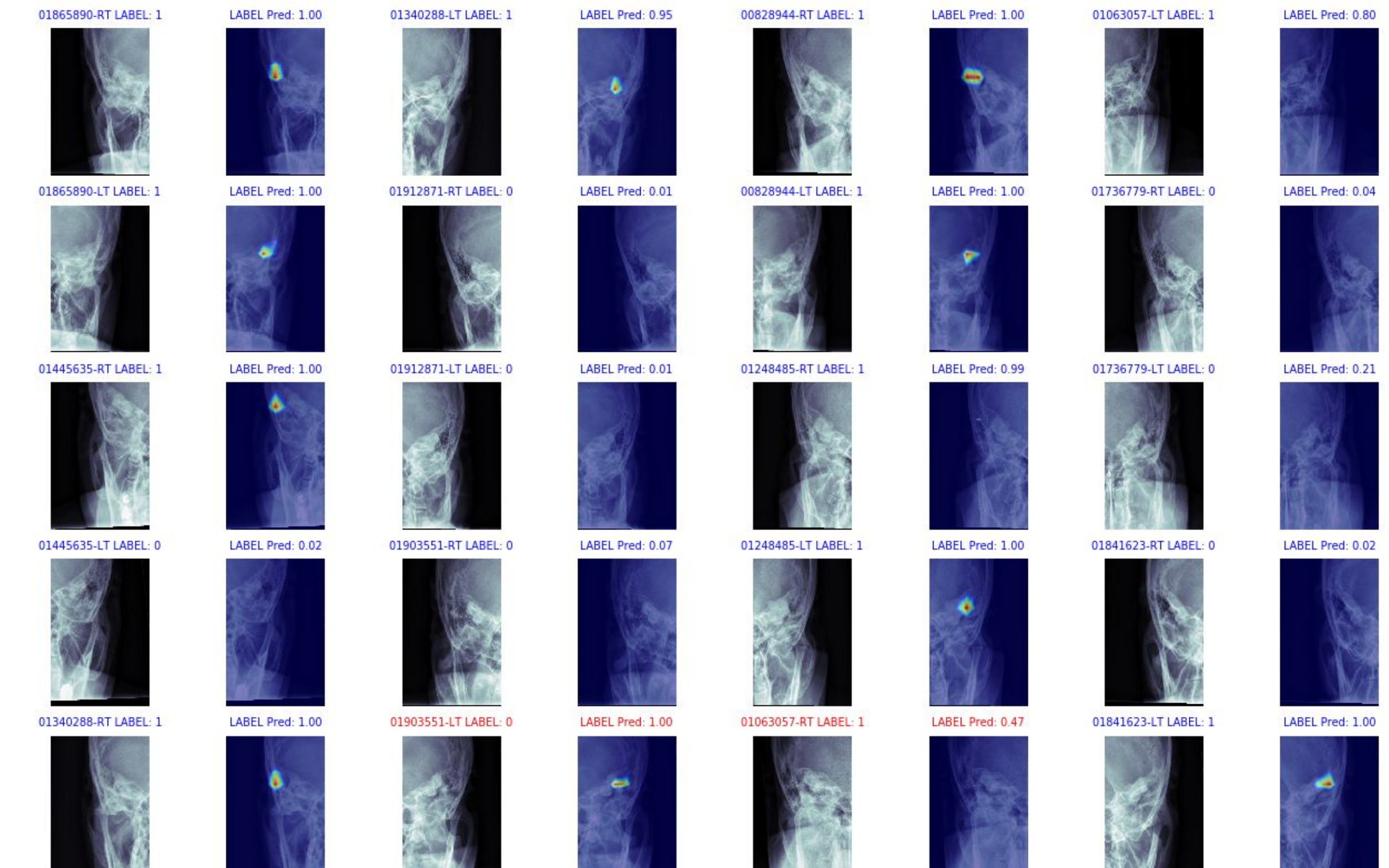

## Slide 10
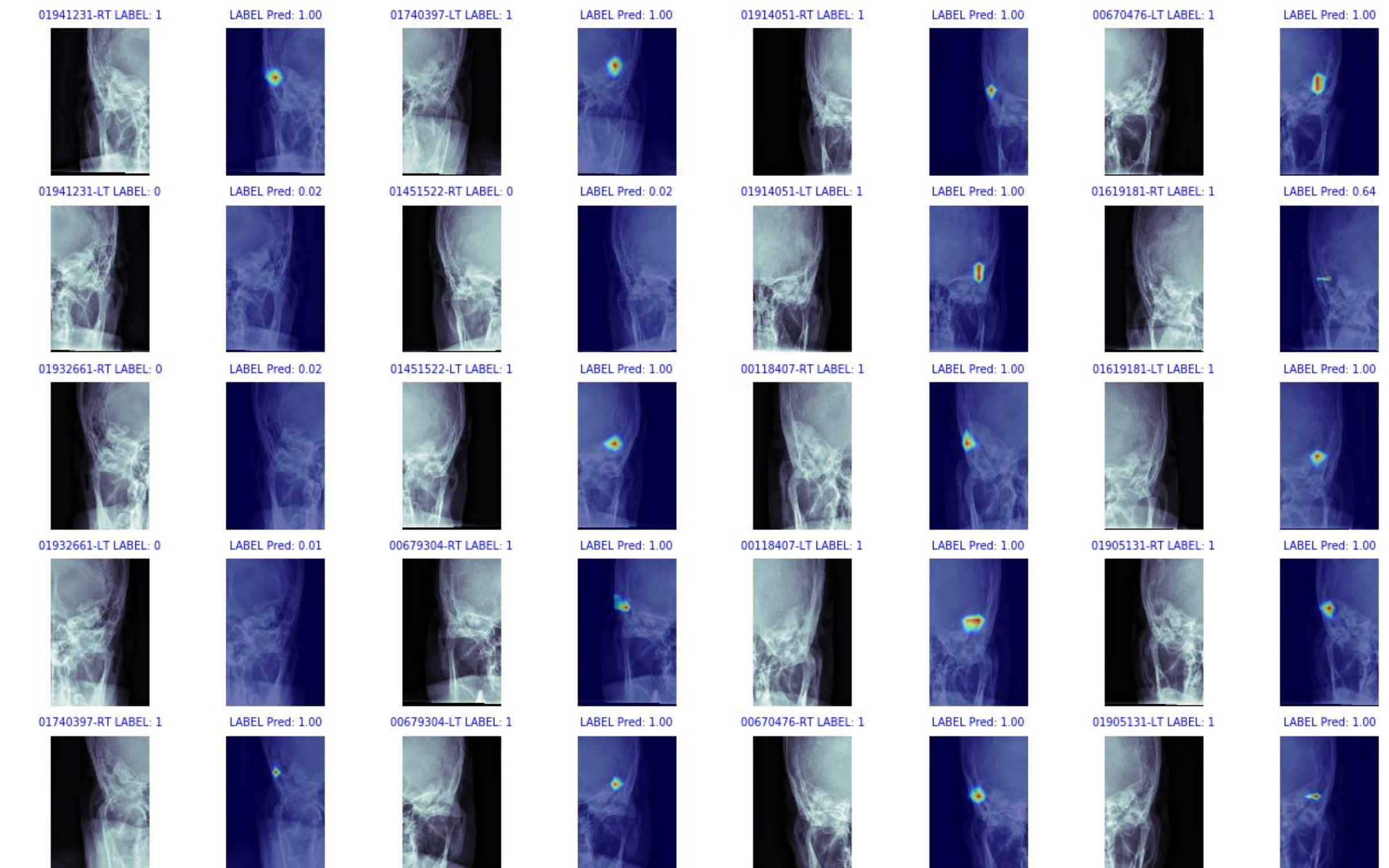

## Slide 11
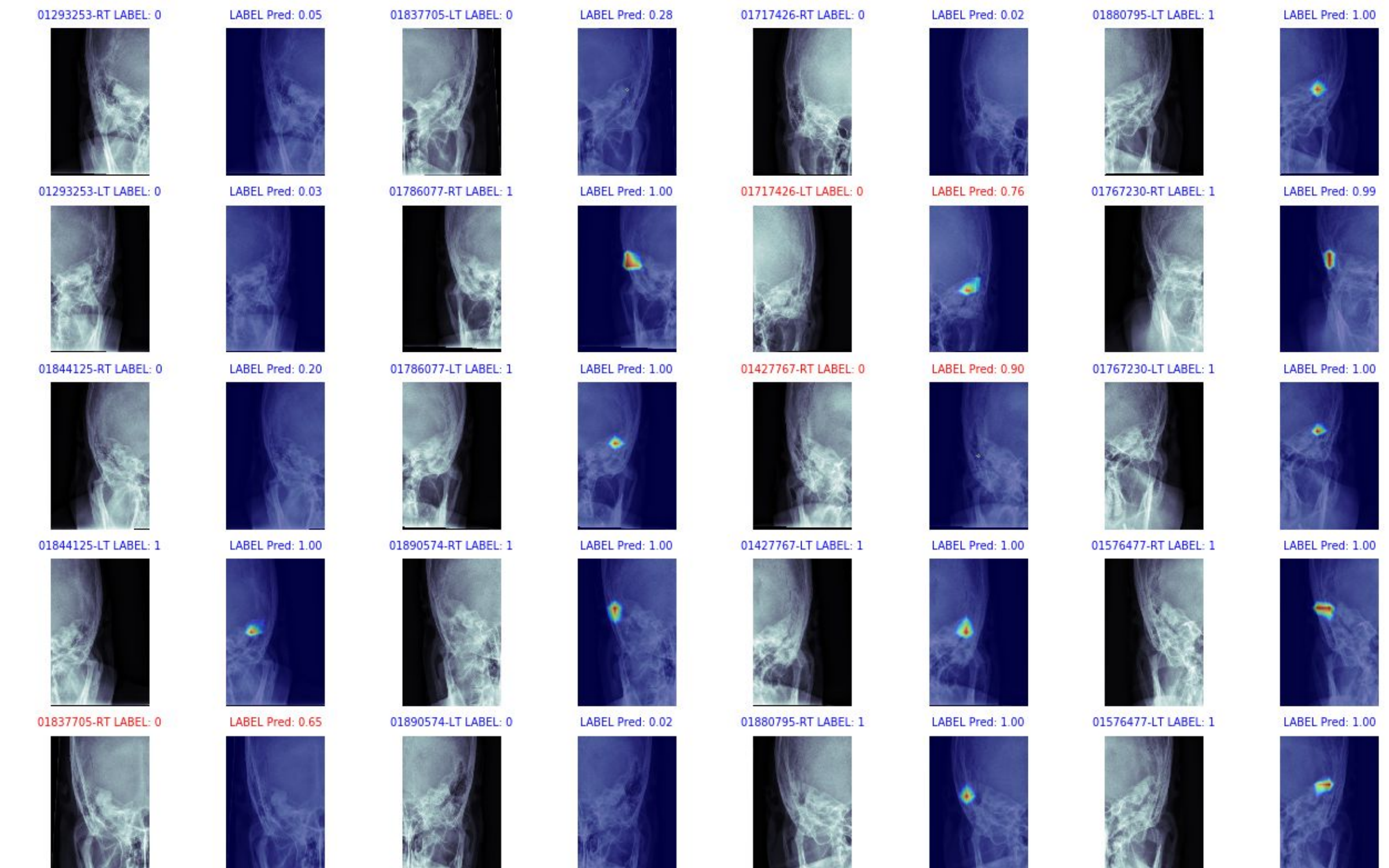

## Slide 12
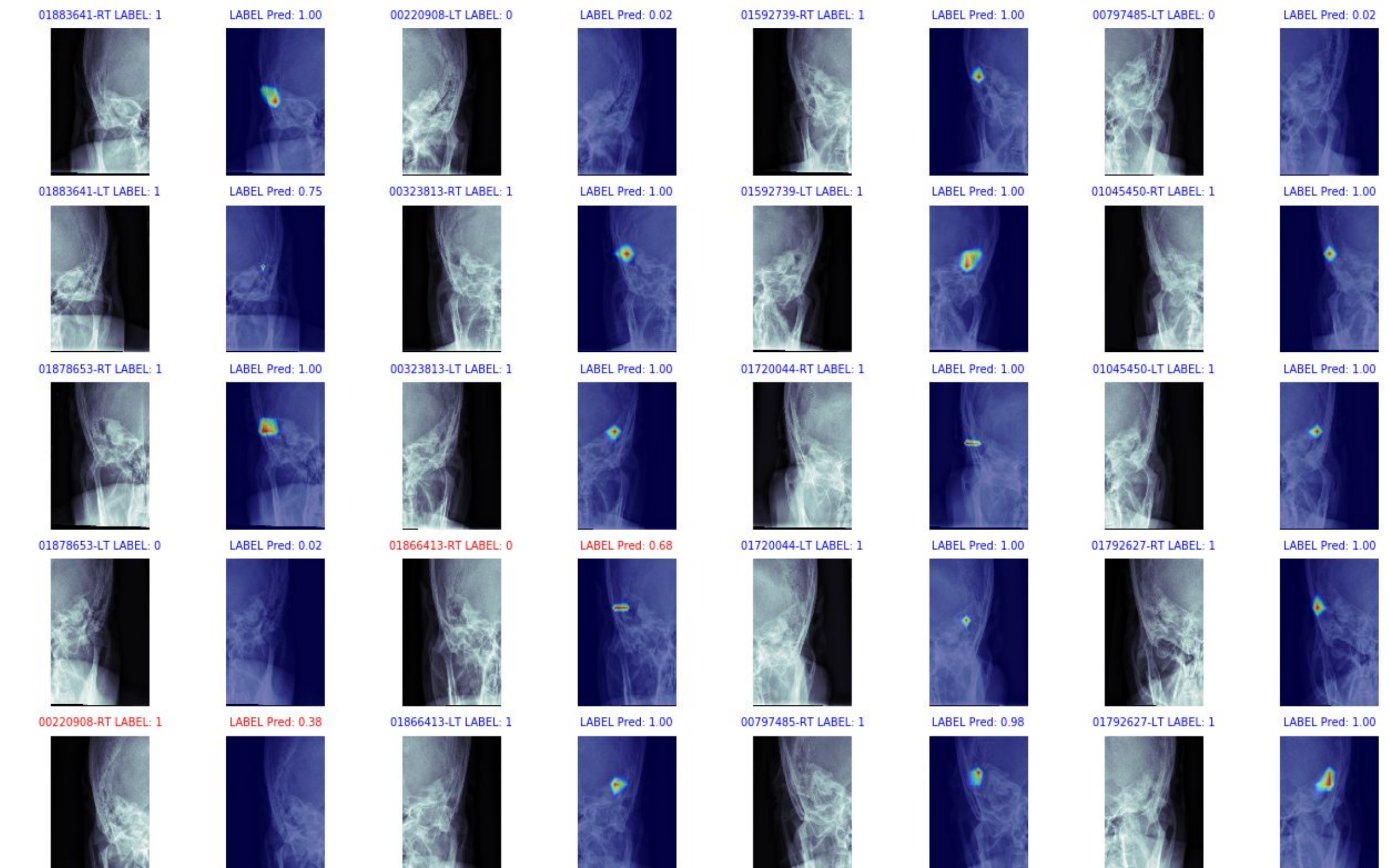

## Slide 13
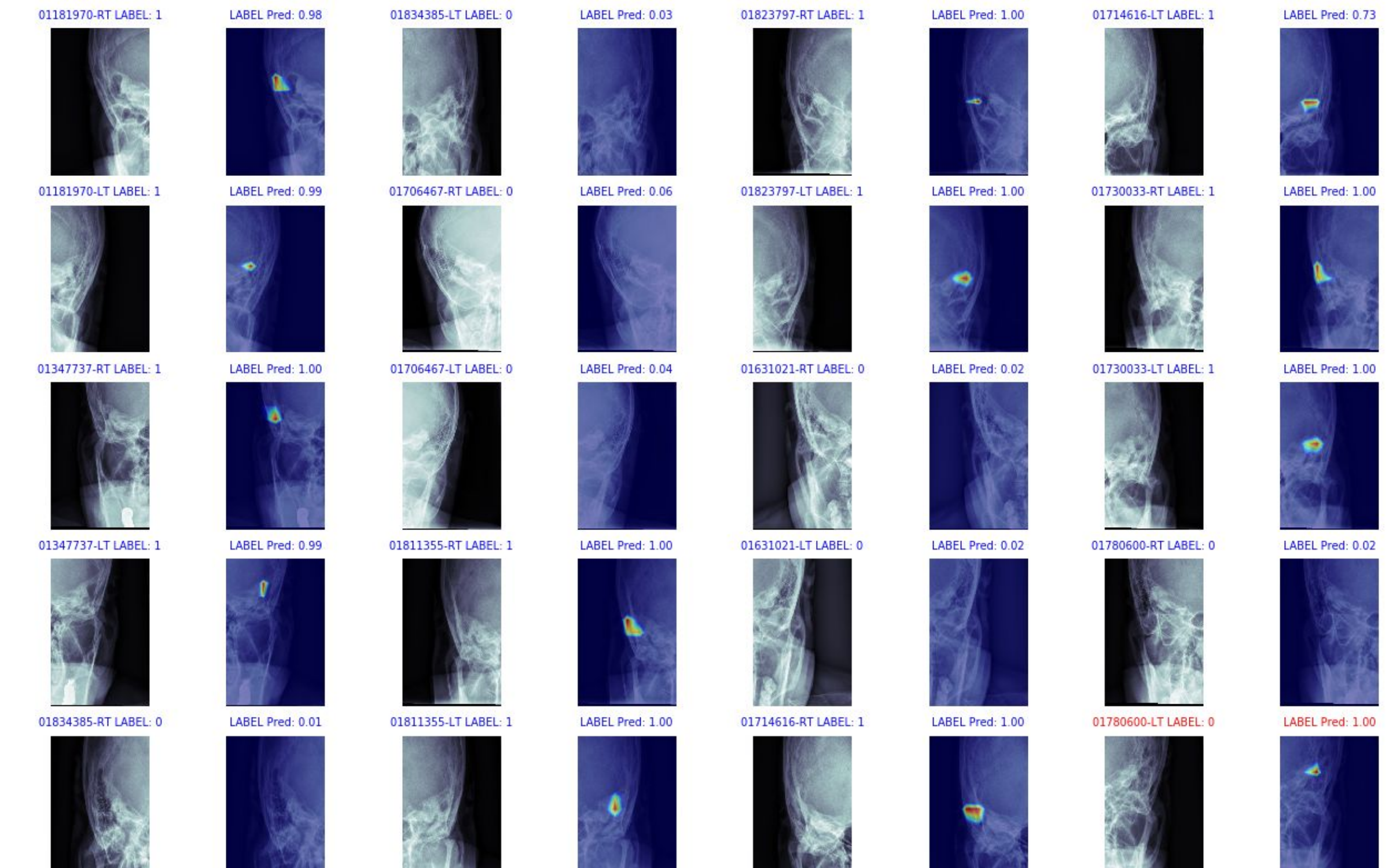

## Slide 14
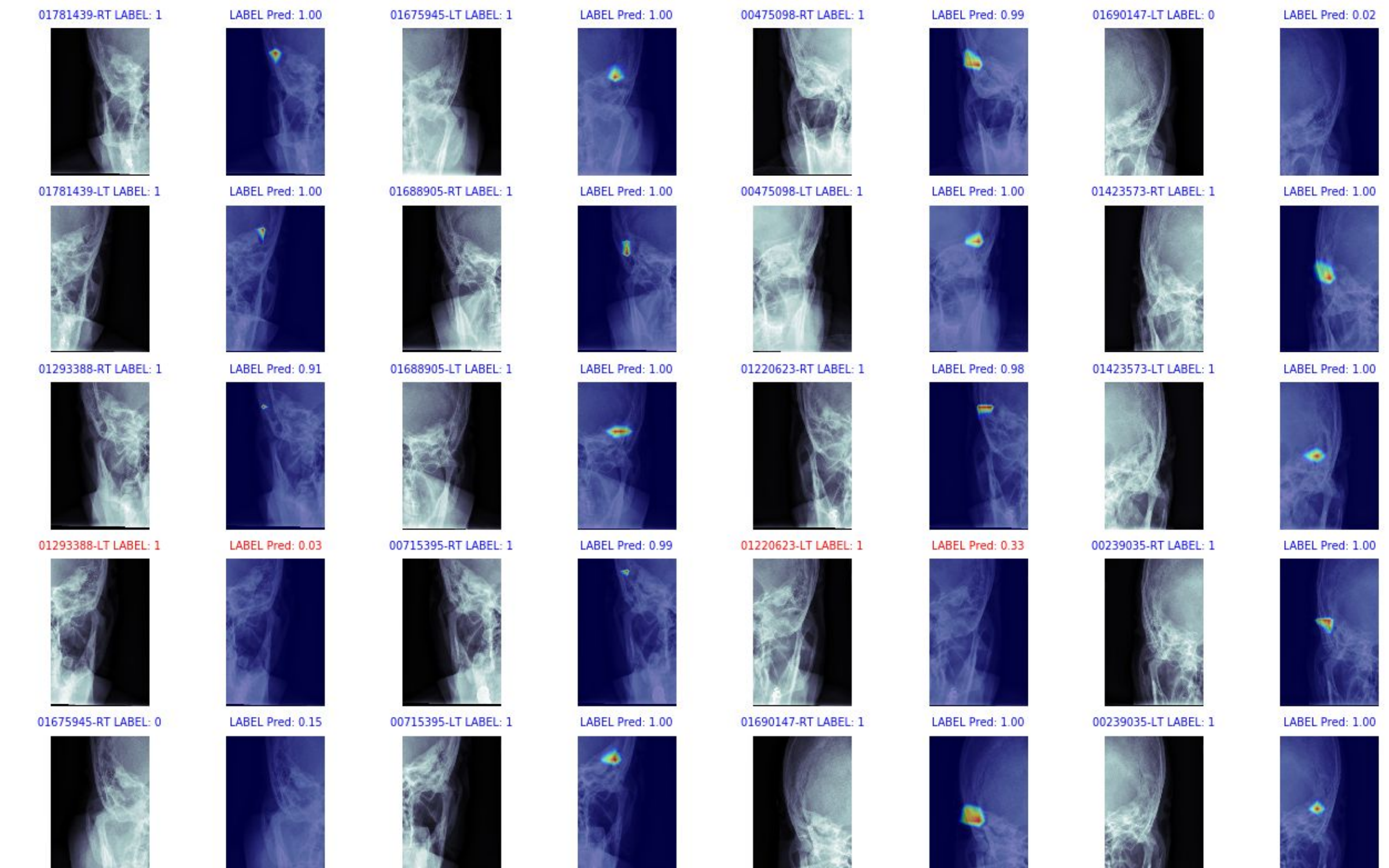

## Slide 15
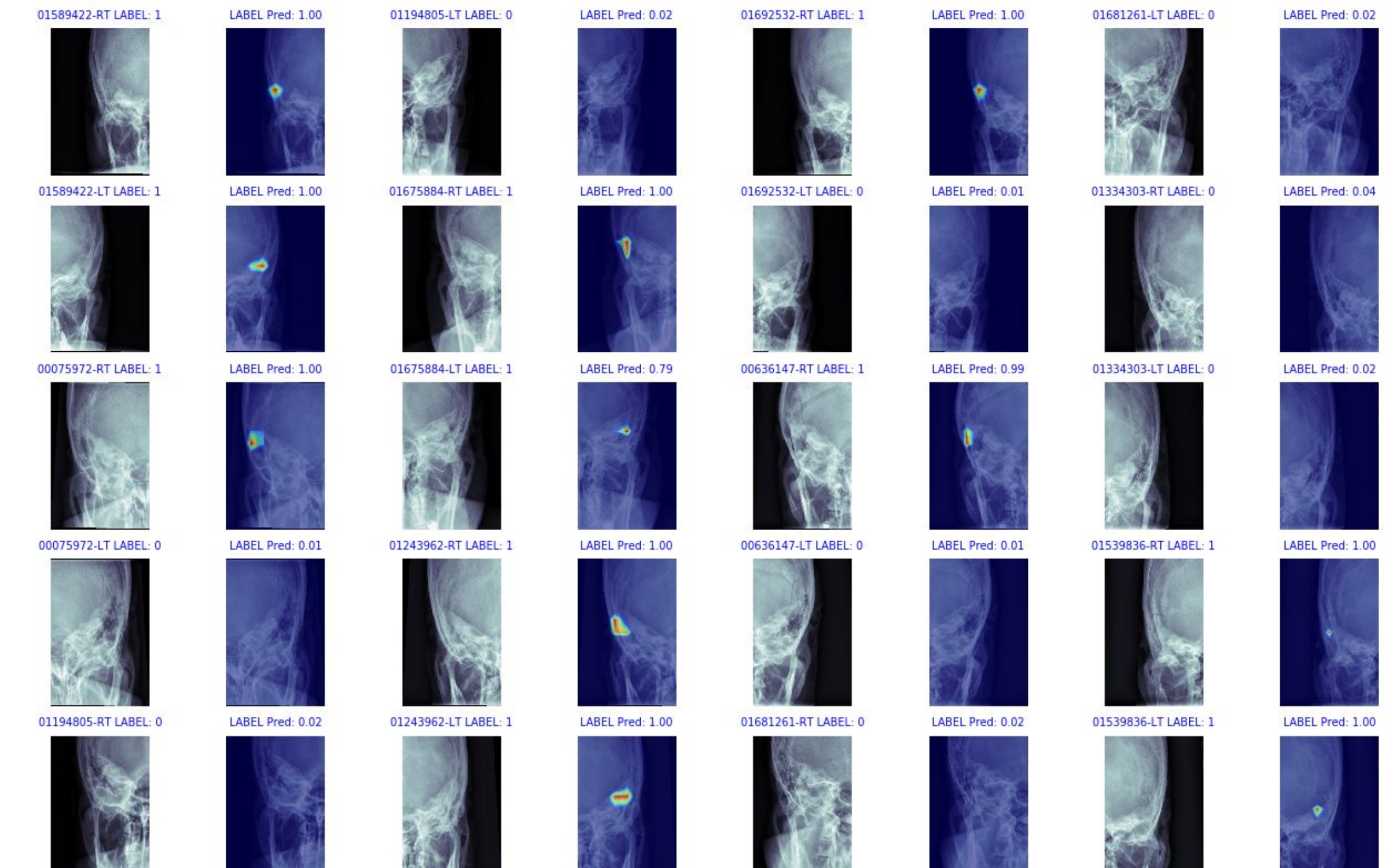

## Slide 16
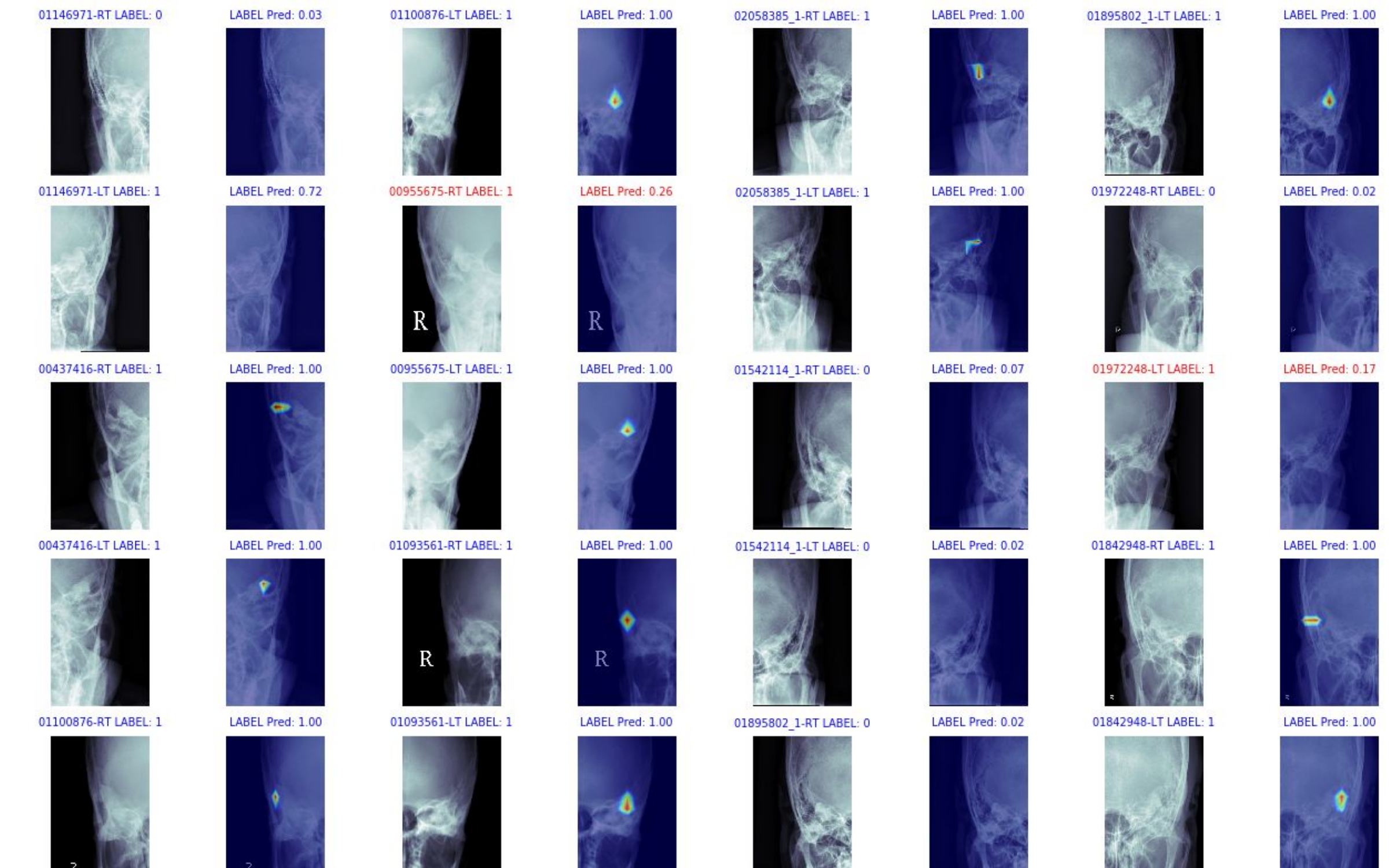

## Slide 17
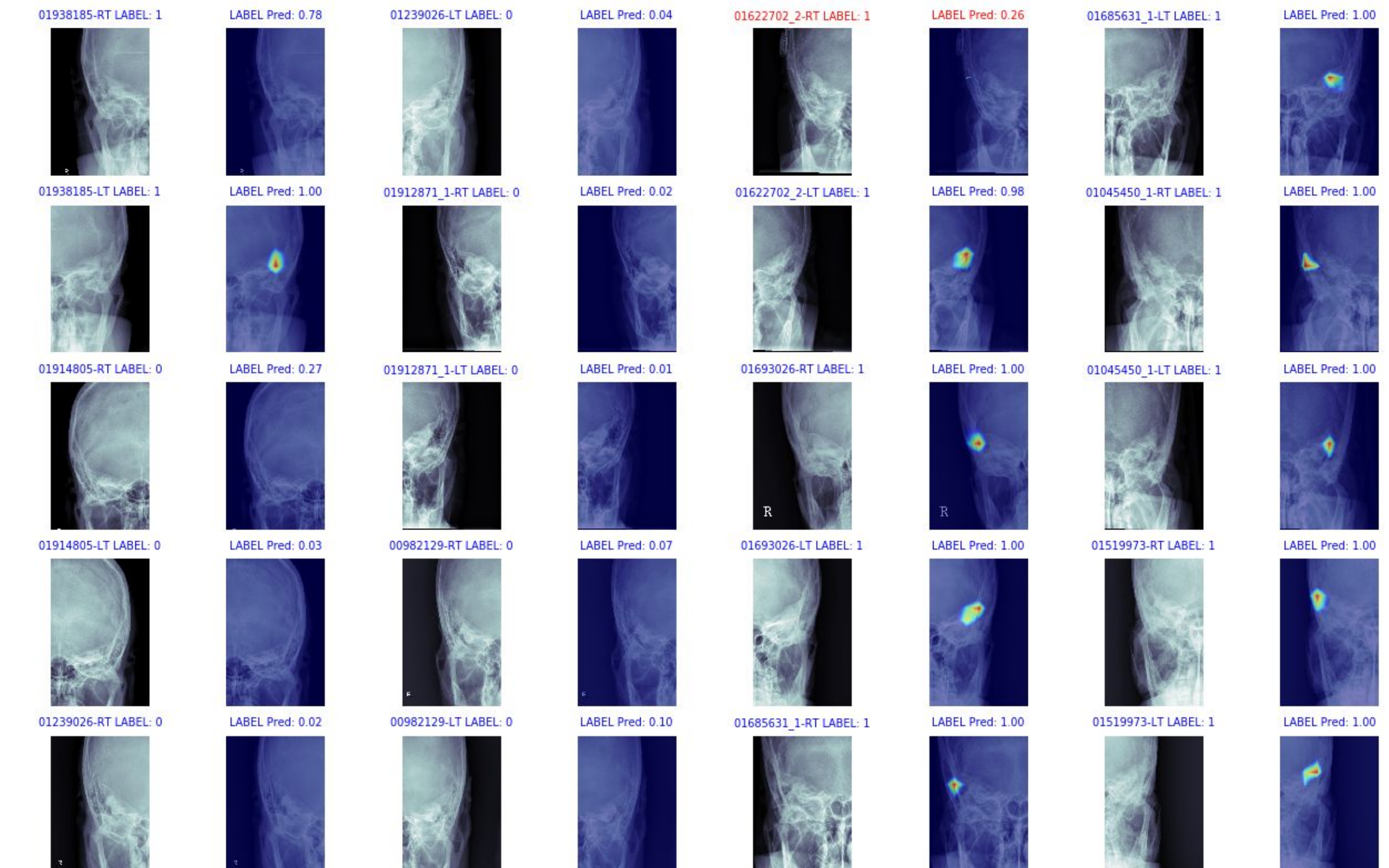

## Slide 18
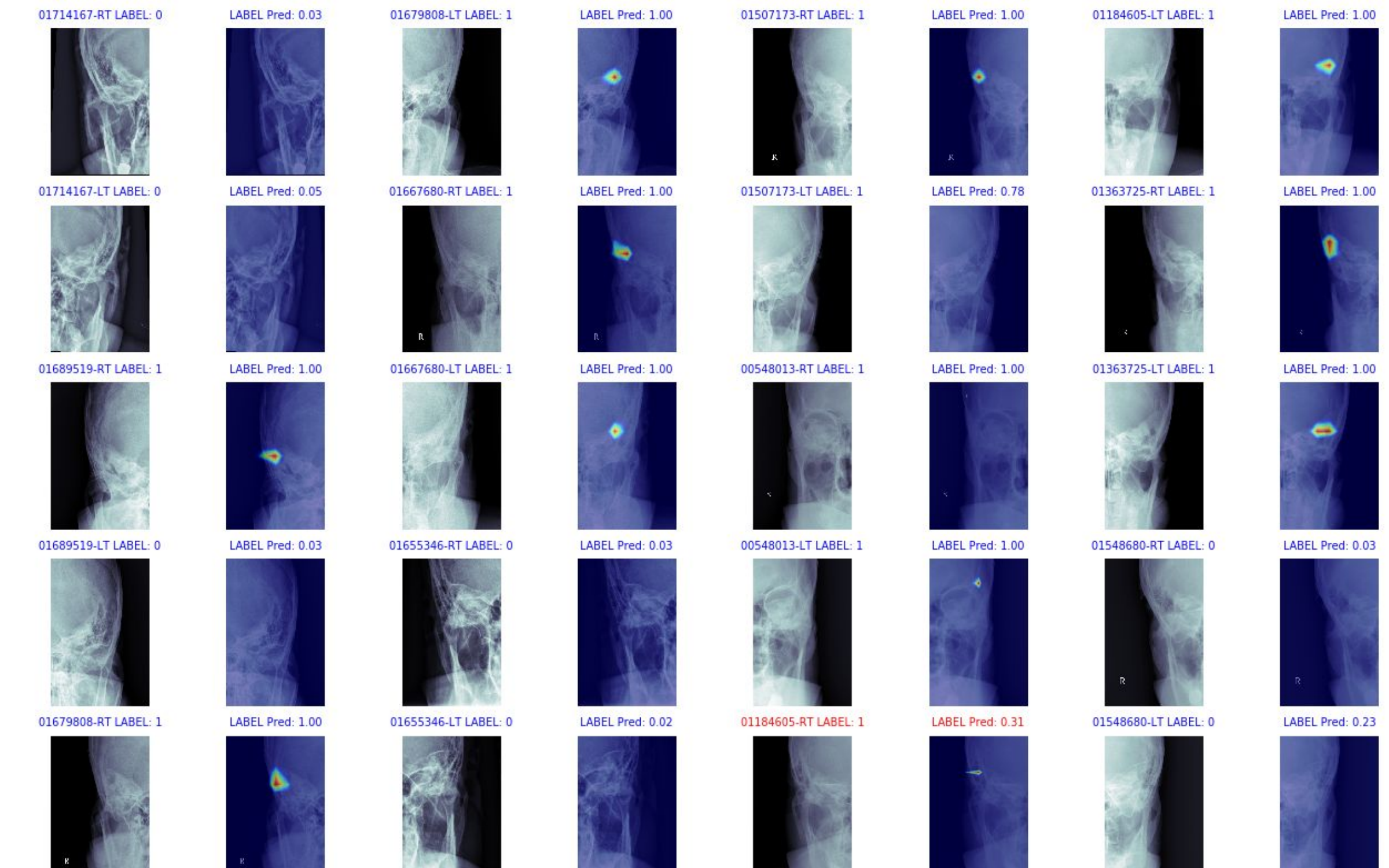

## Slide 19
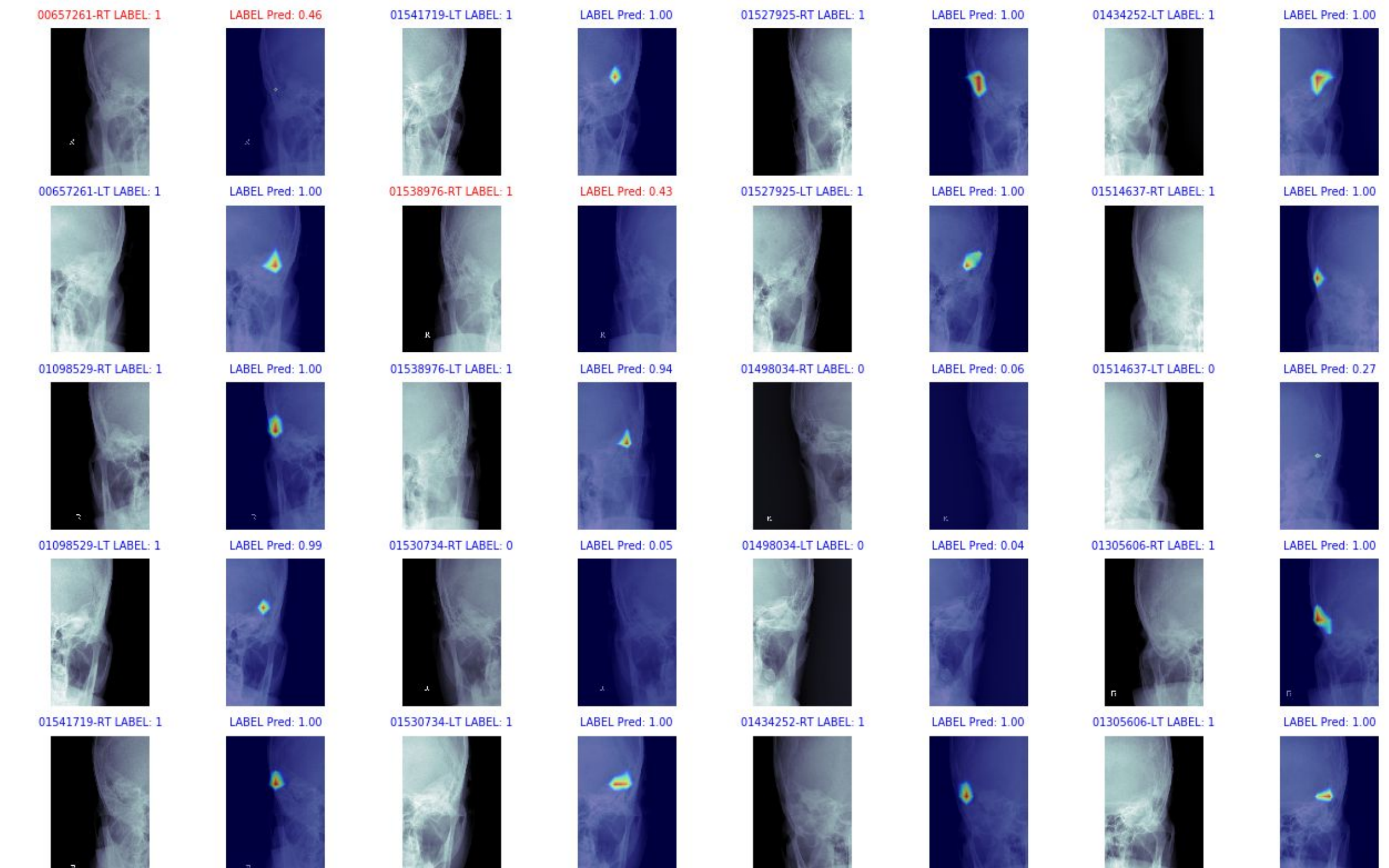

## Slide 20
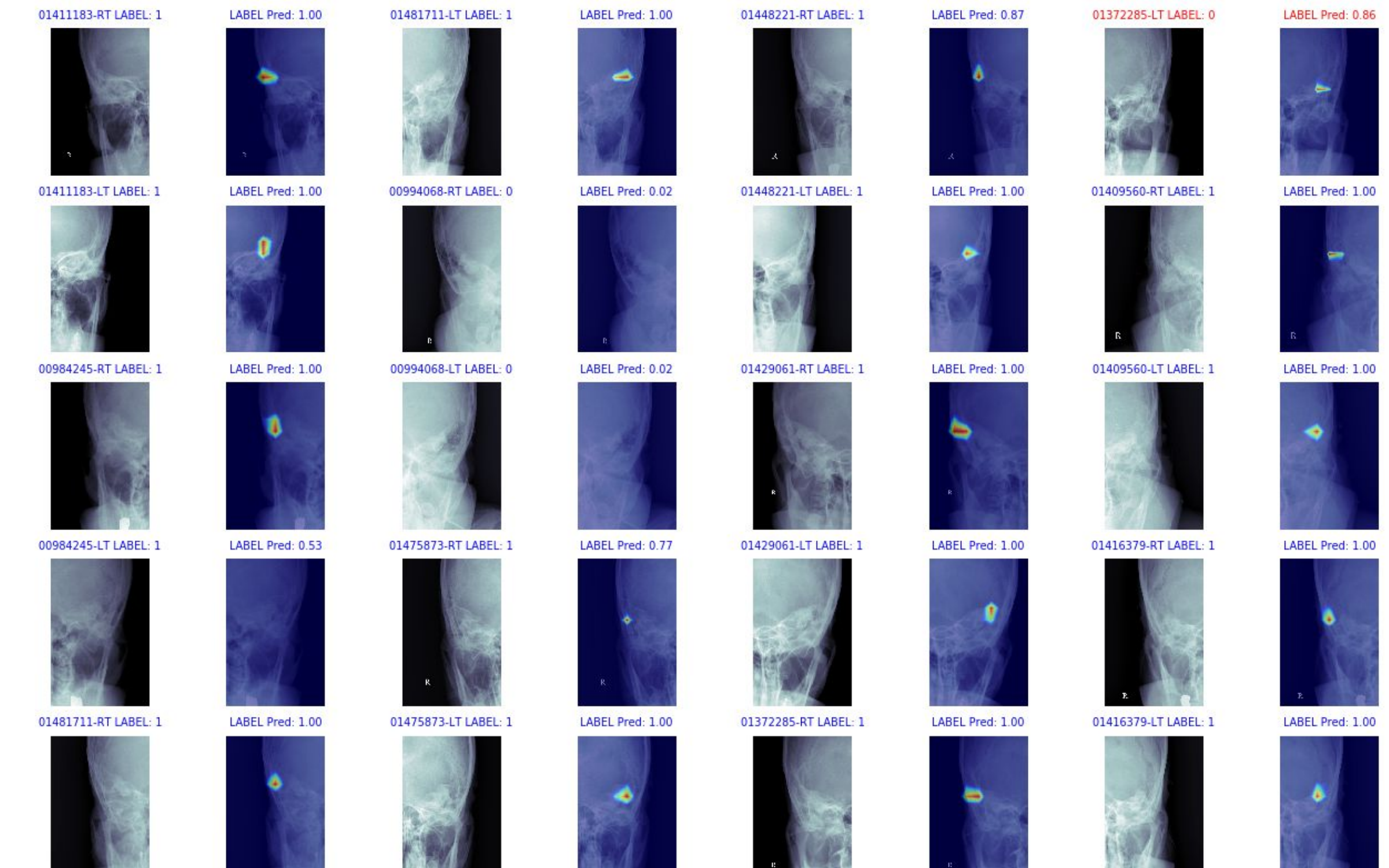

## Slide 21
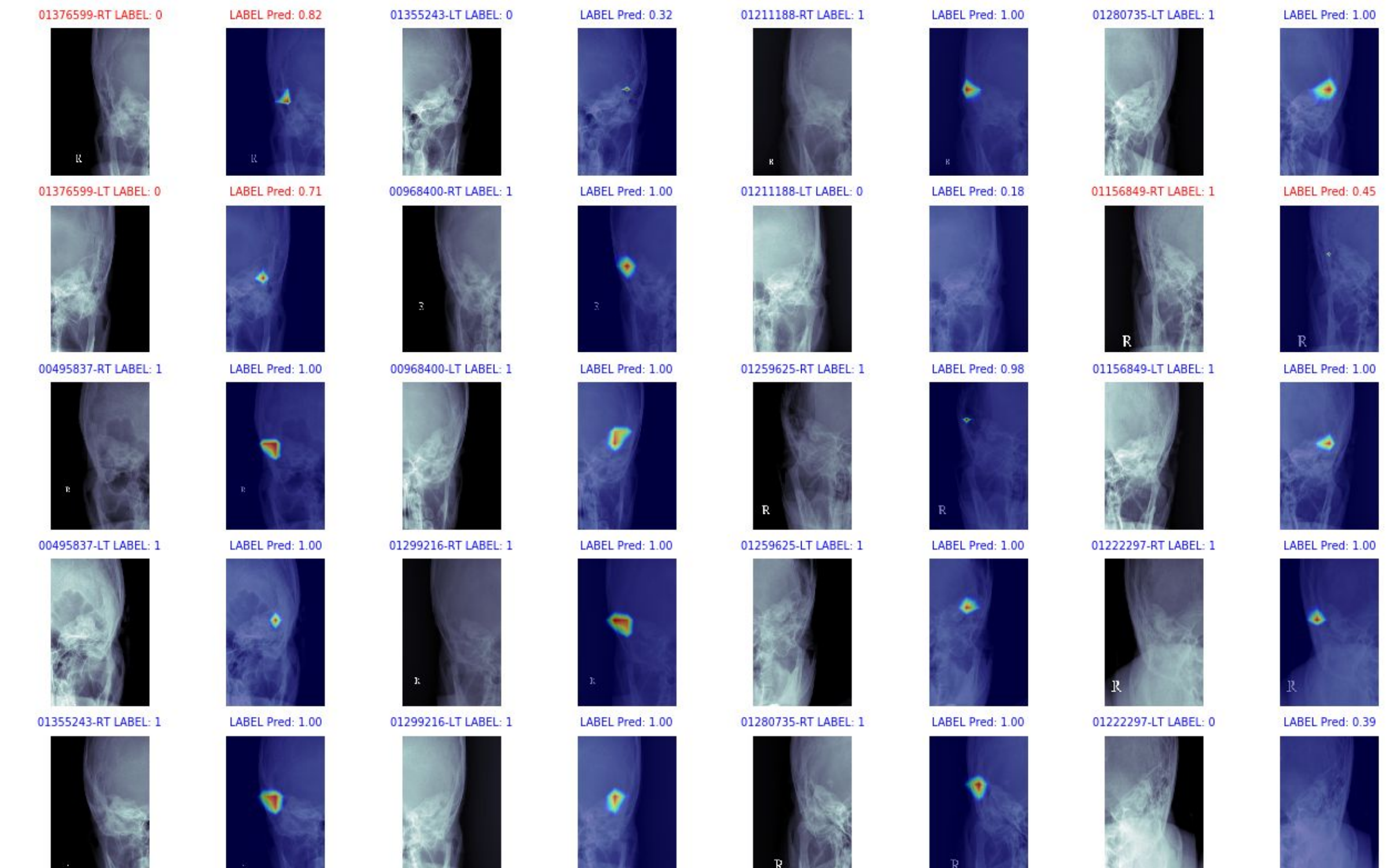

## Slide 22
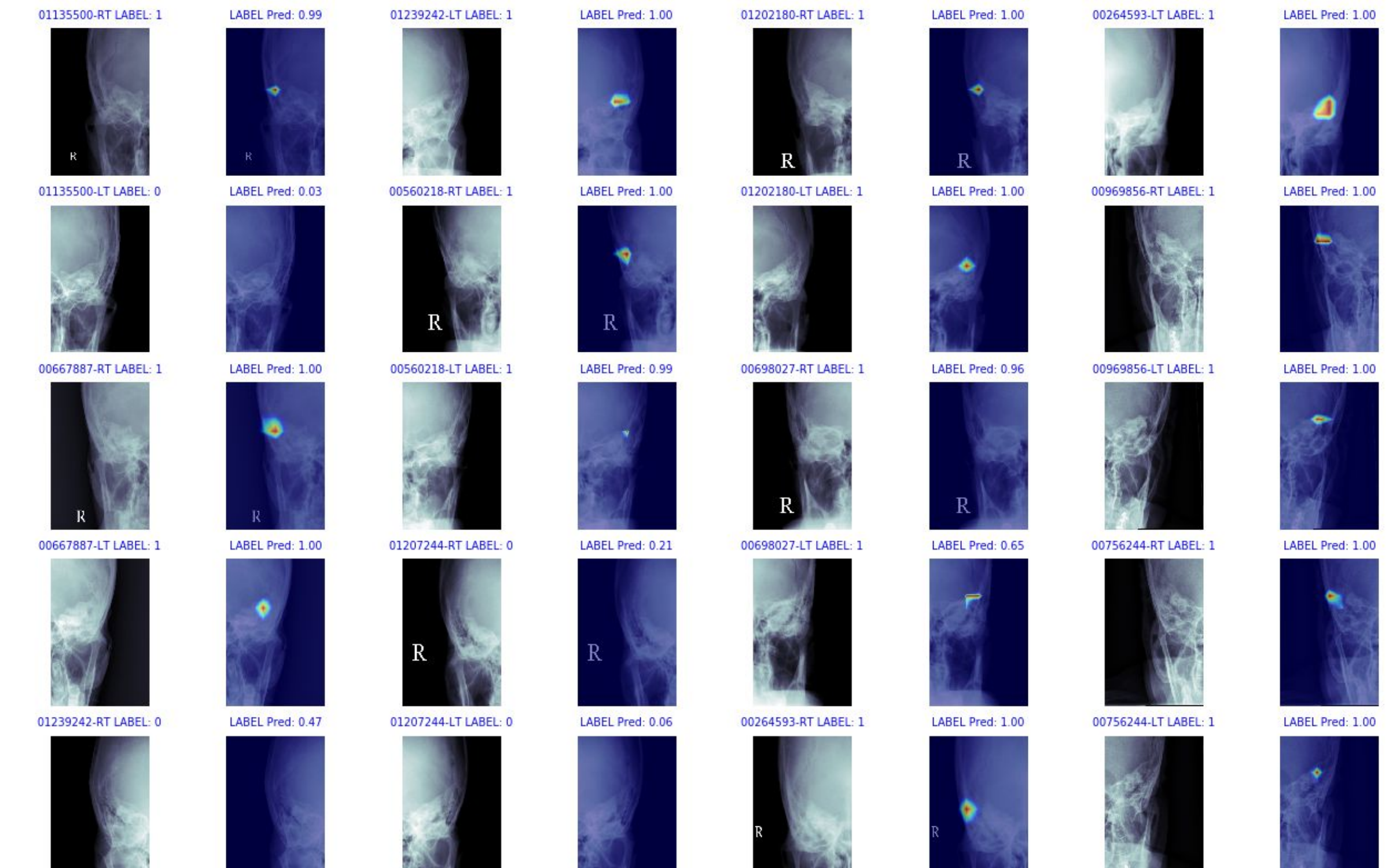

## Slide 23
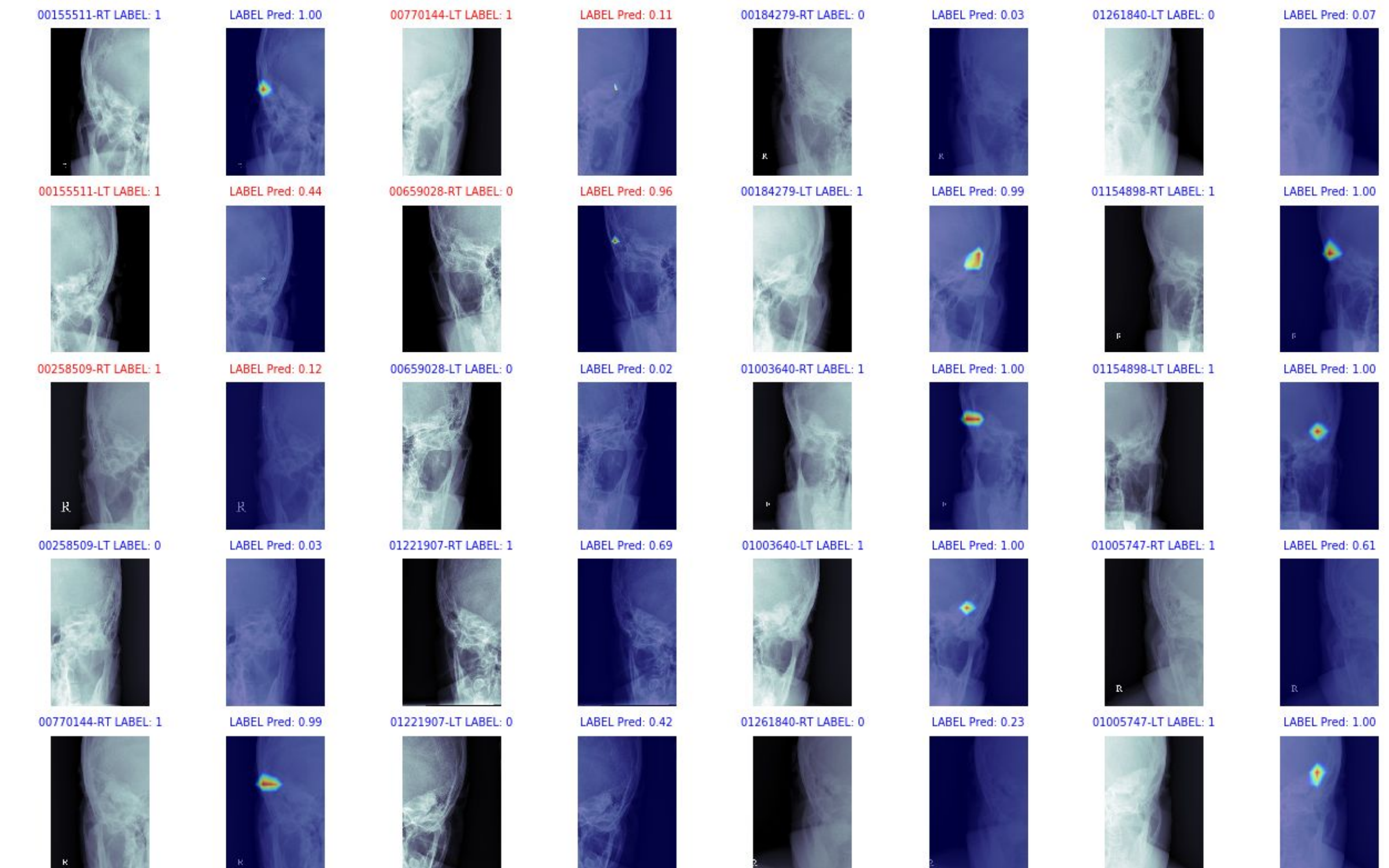

## Slide 24
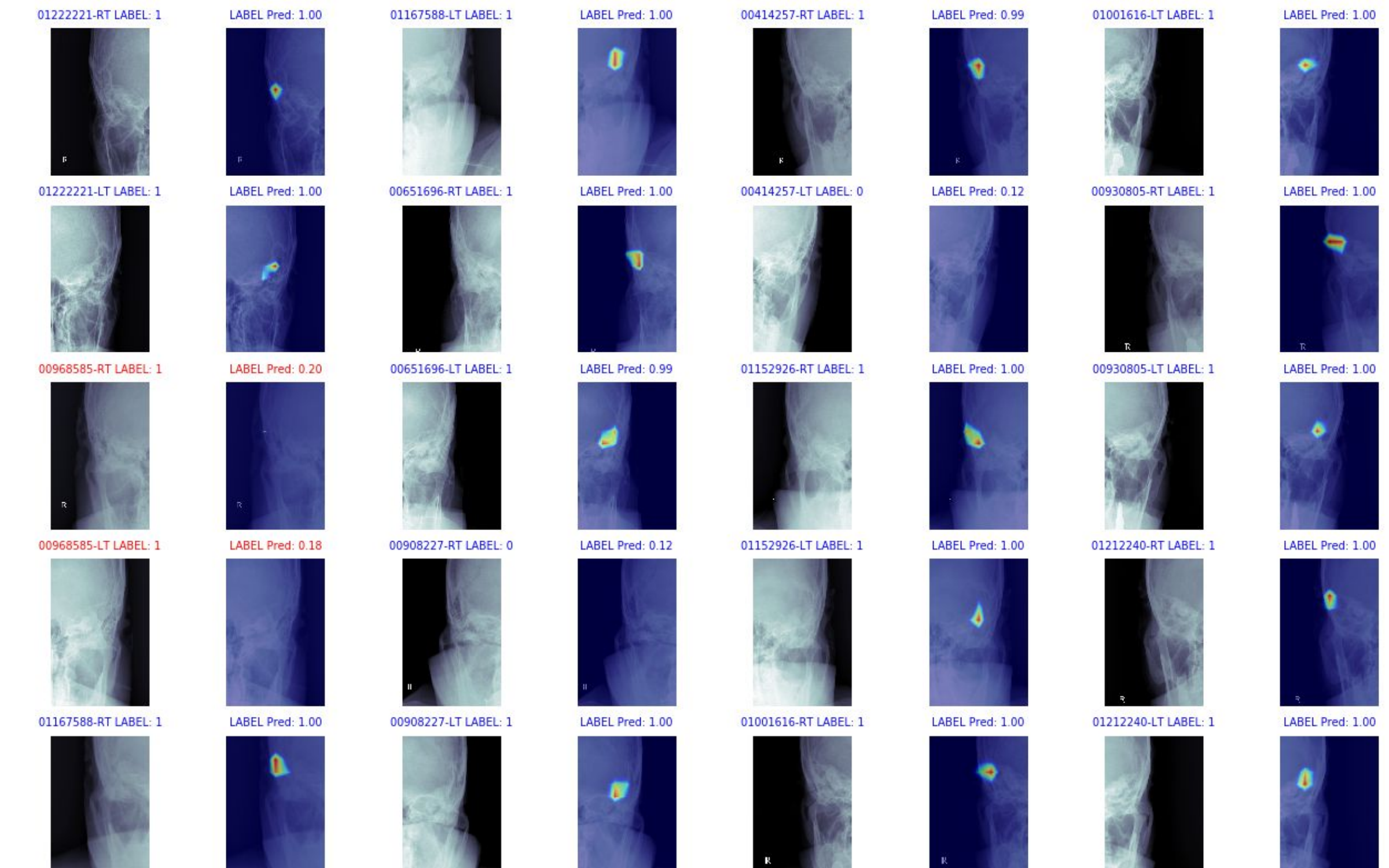

## Slide 25
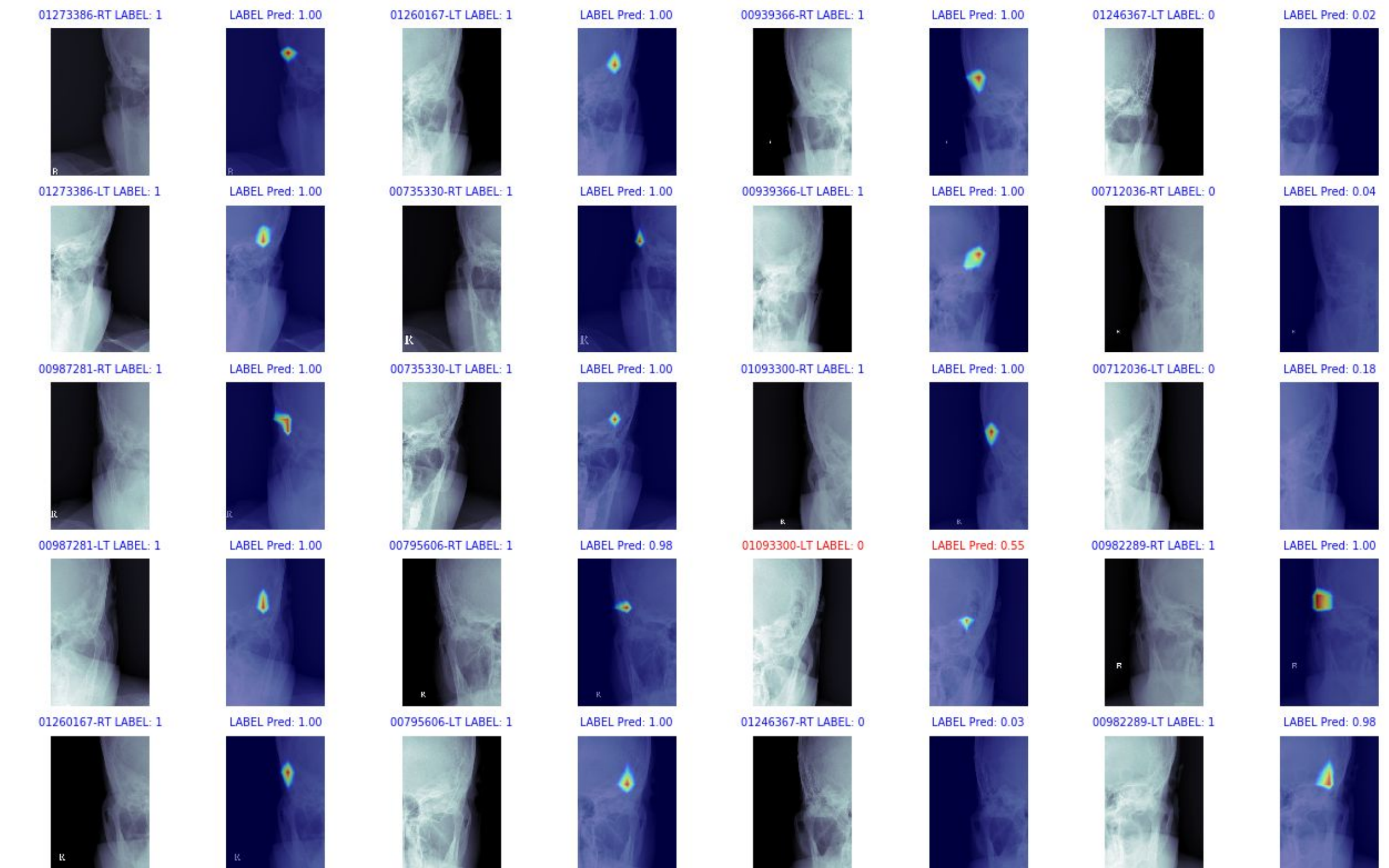

## Slide 26
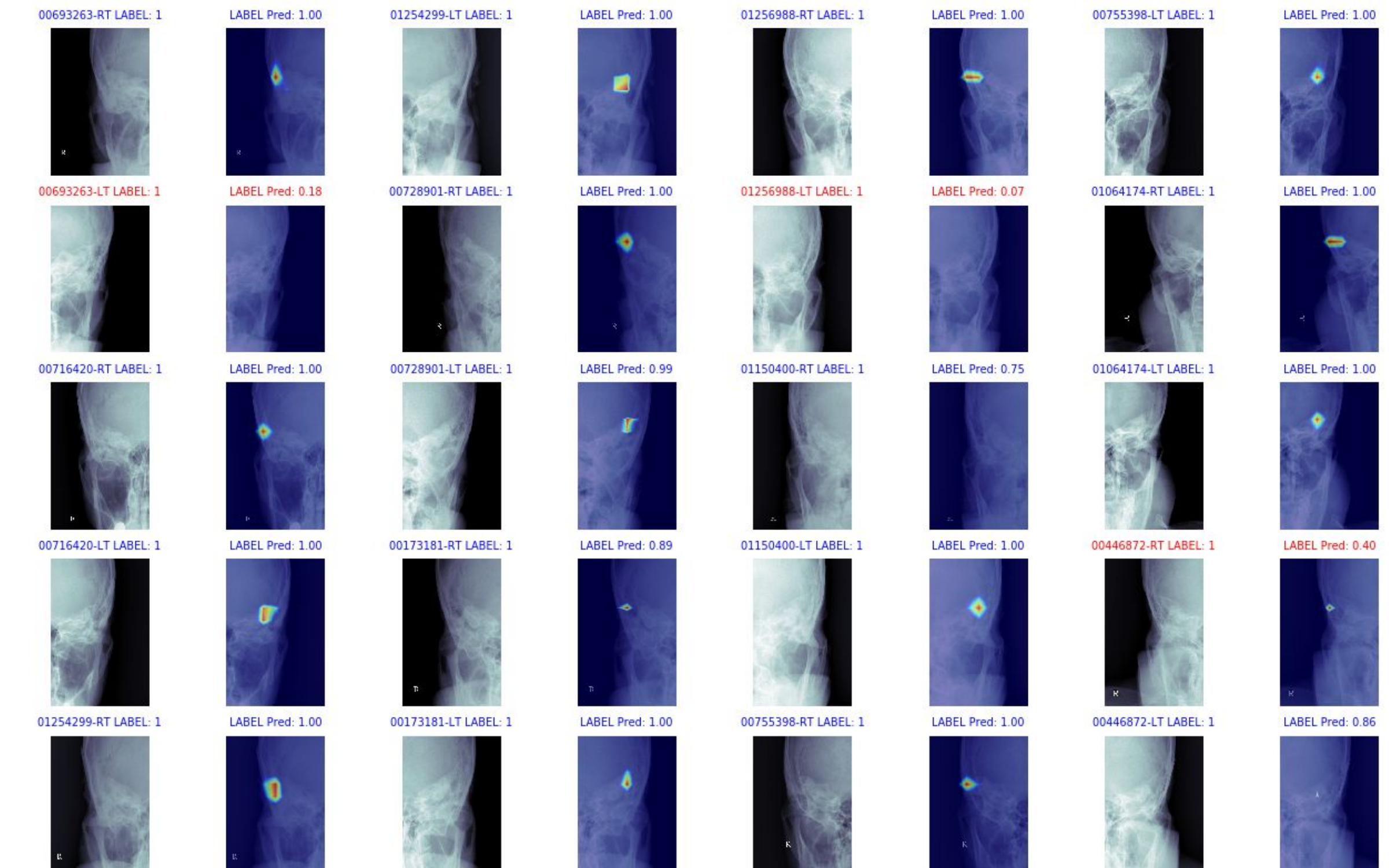

## Slide 27
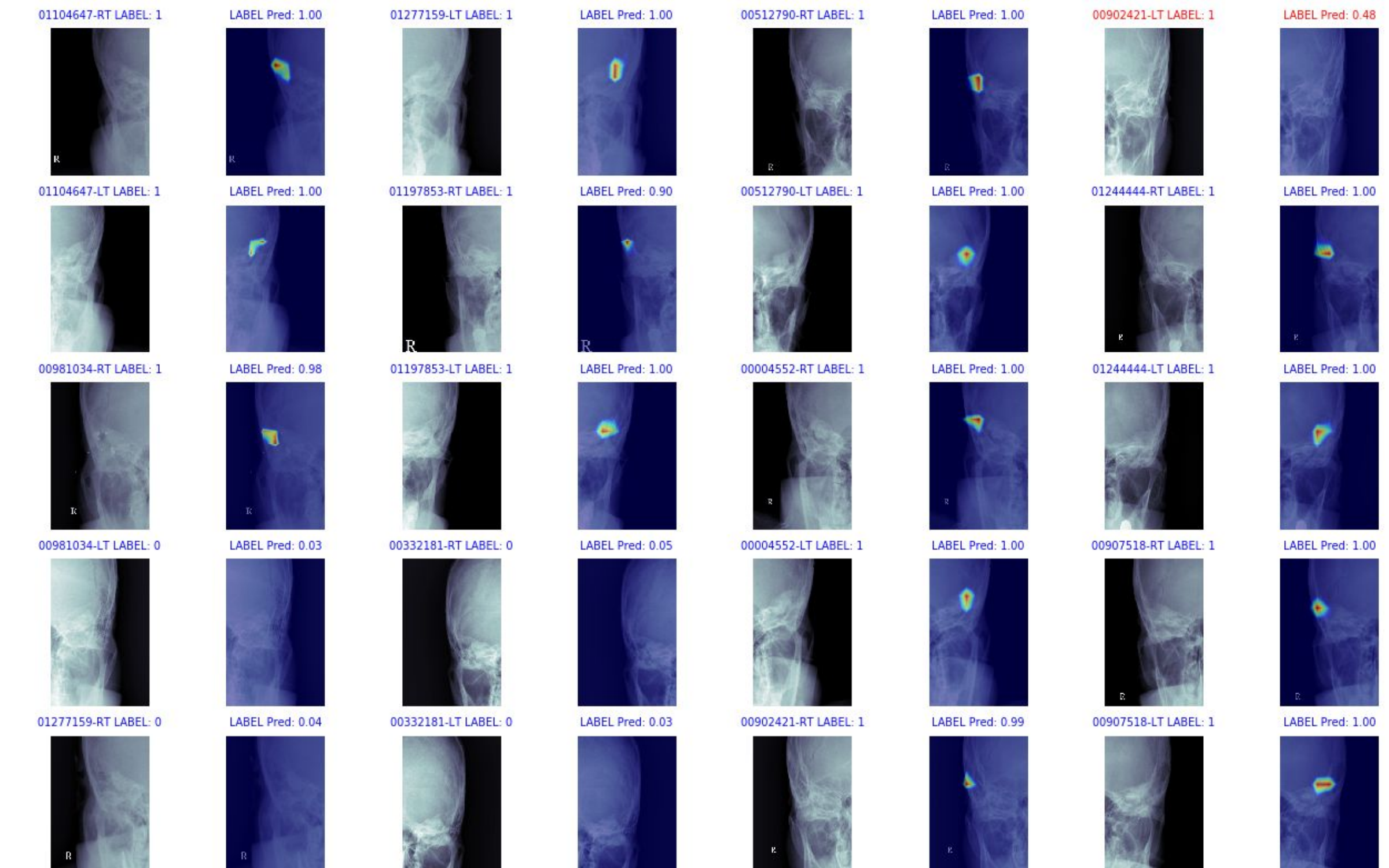

## Slide 28
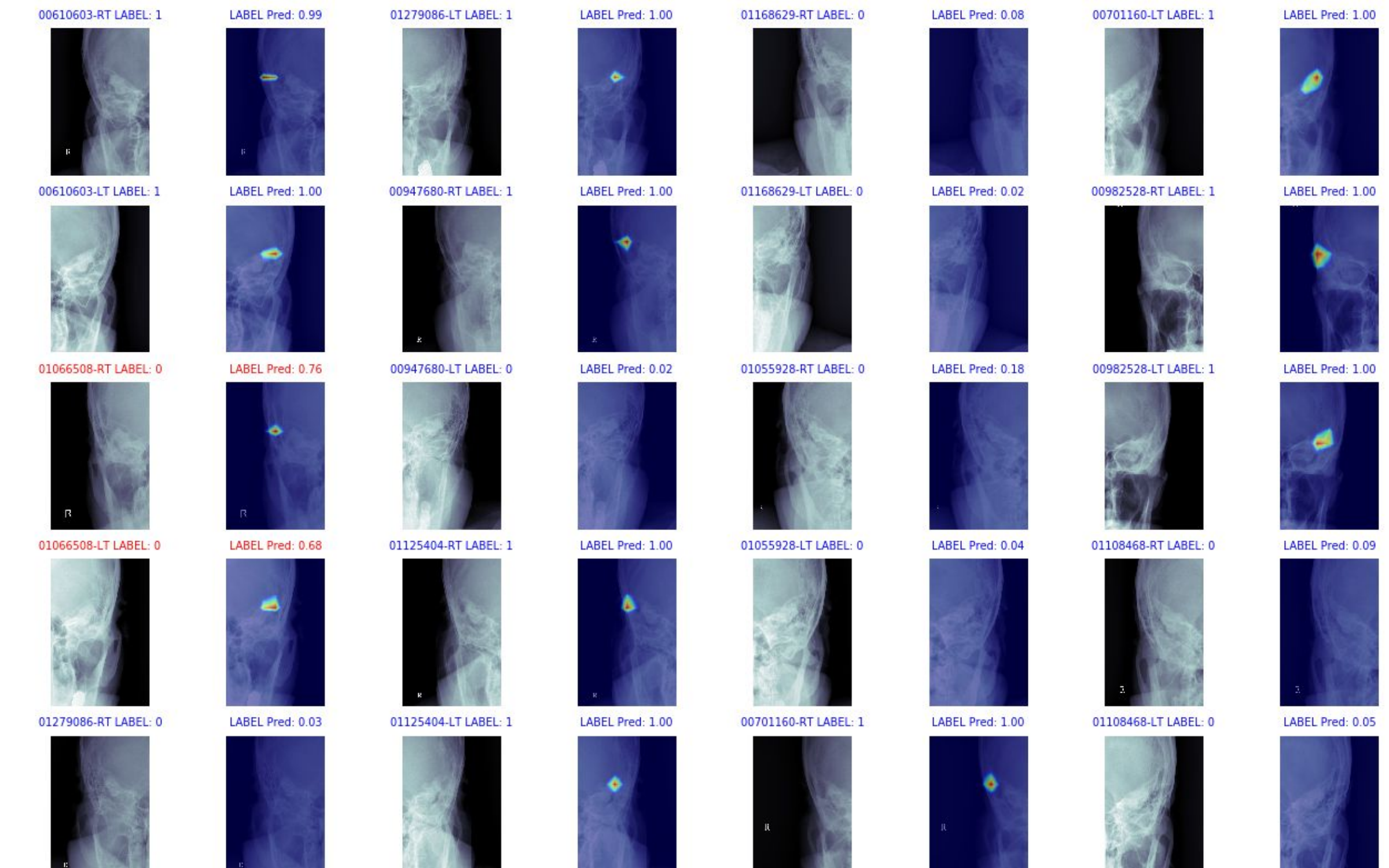

## Slide 29
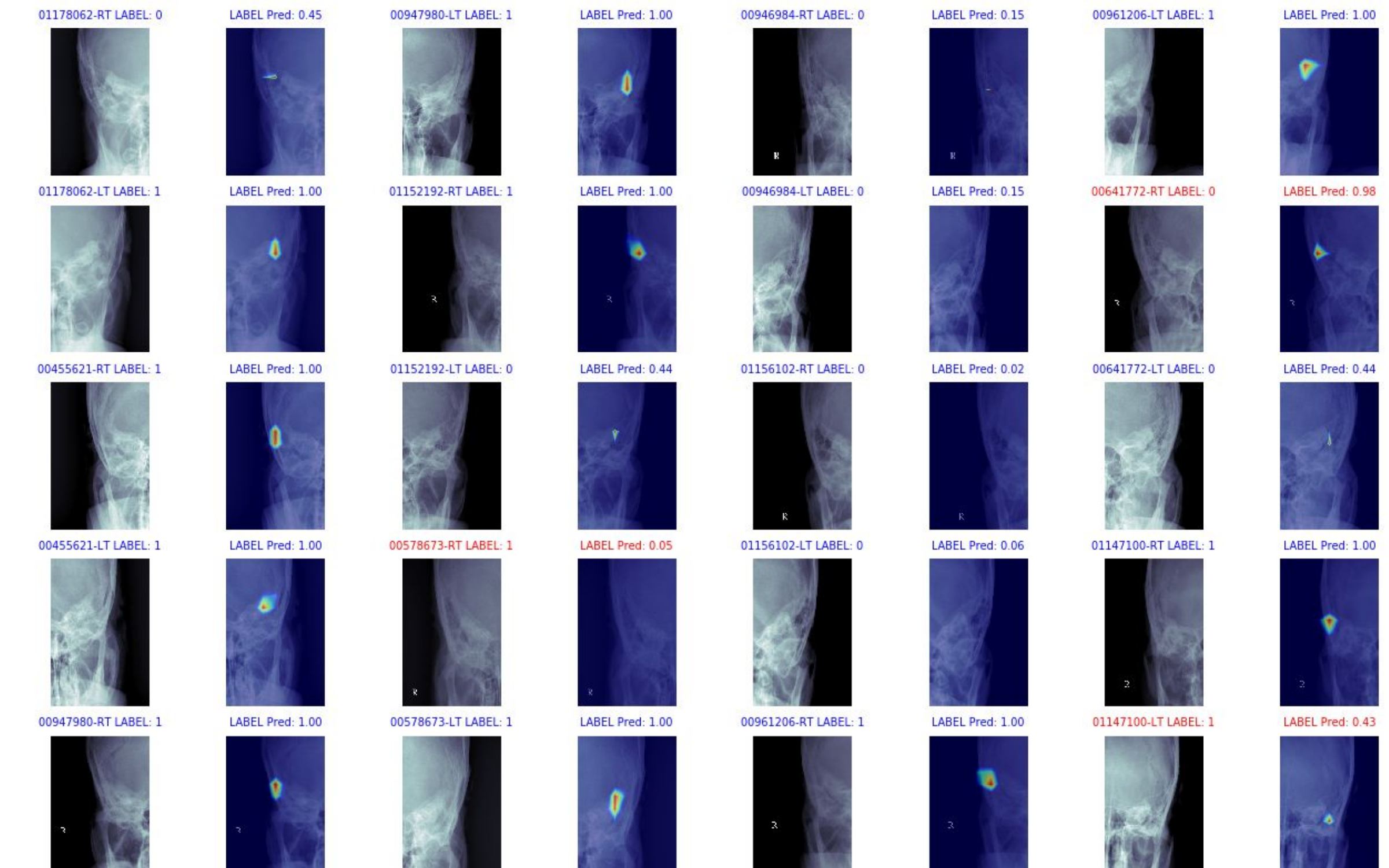

## Slide 30
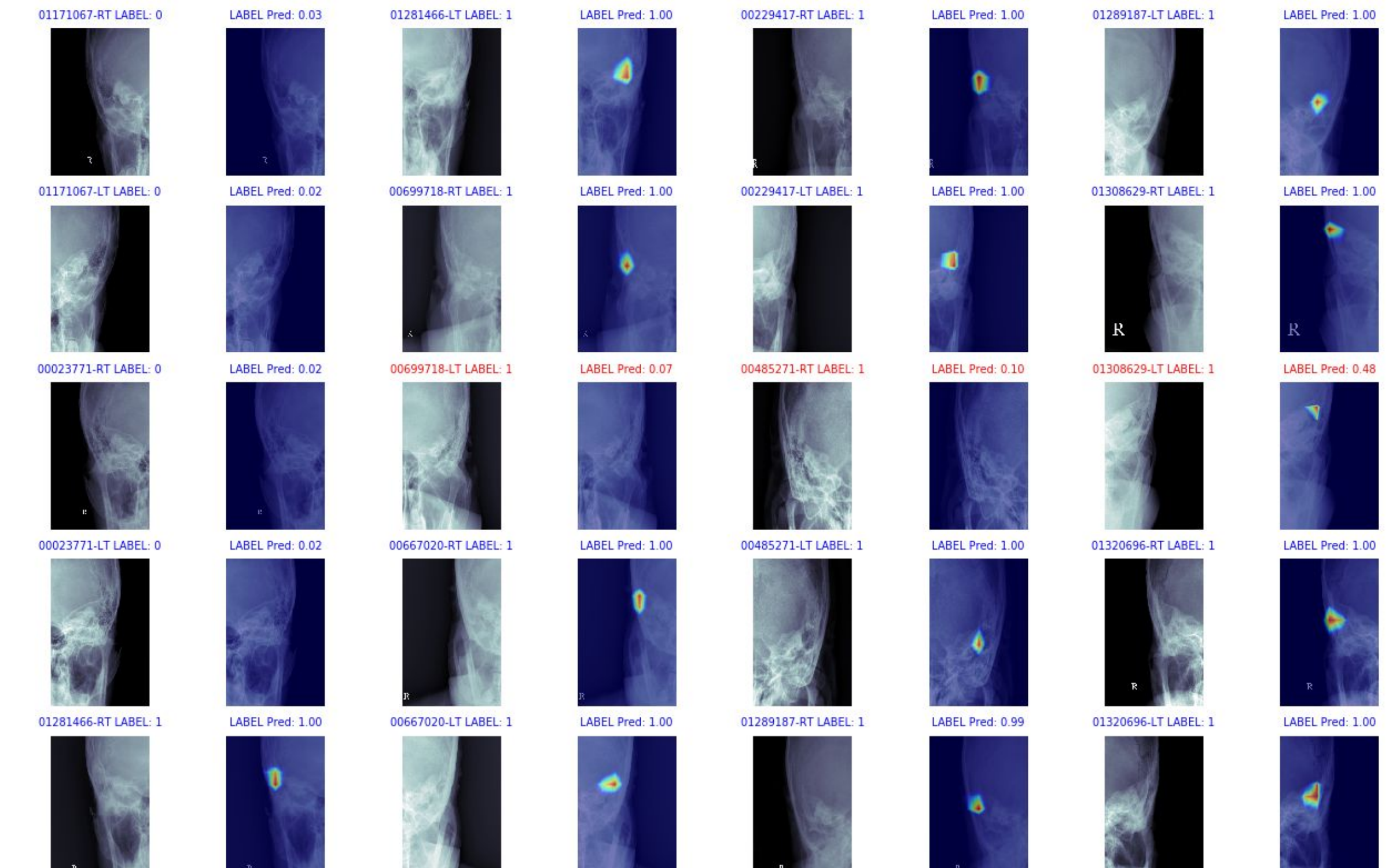

## Slide 31
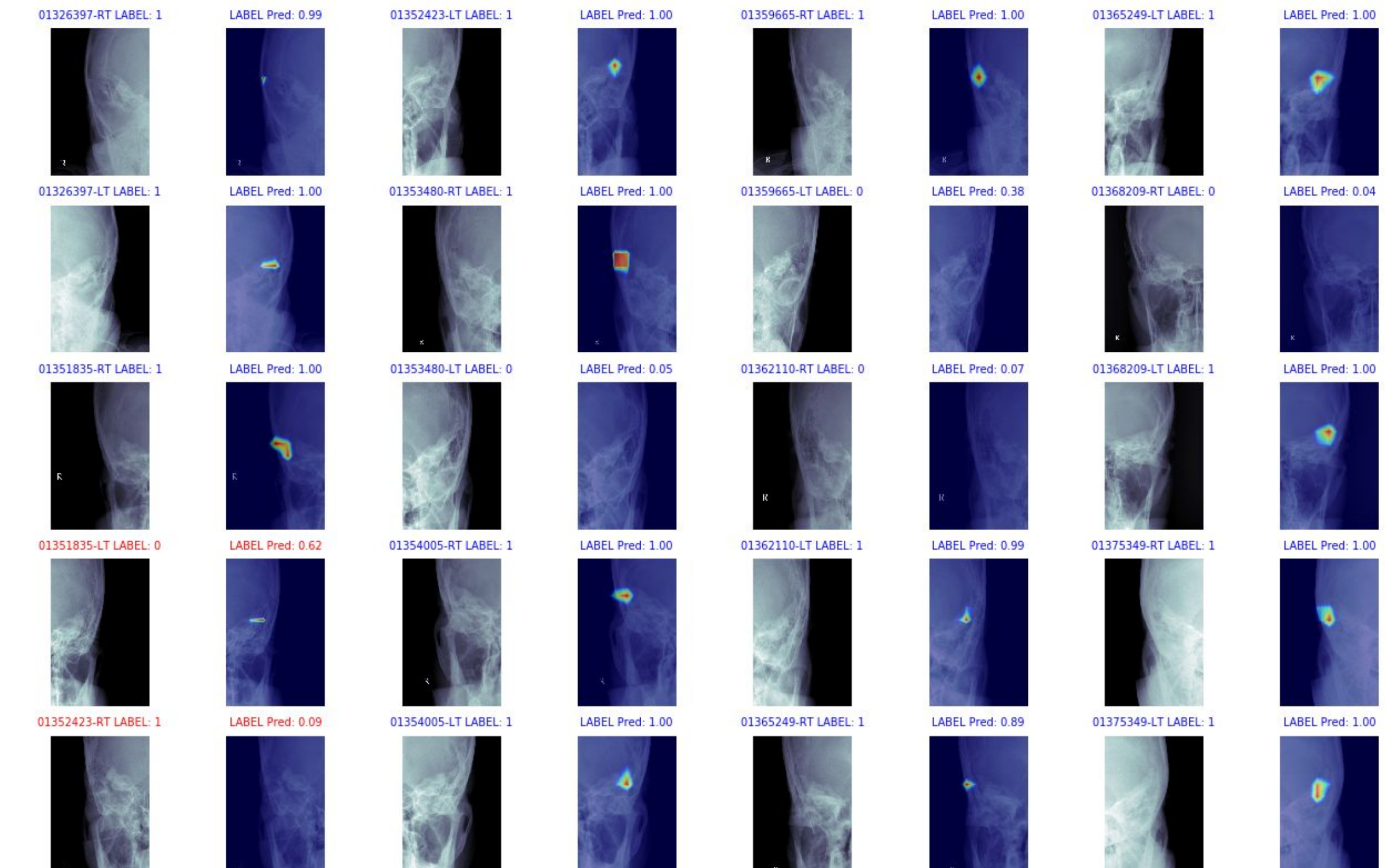

## Slide 32
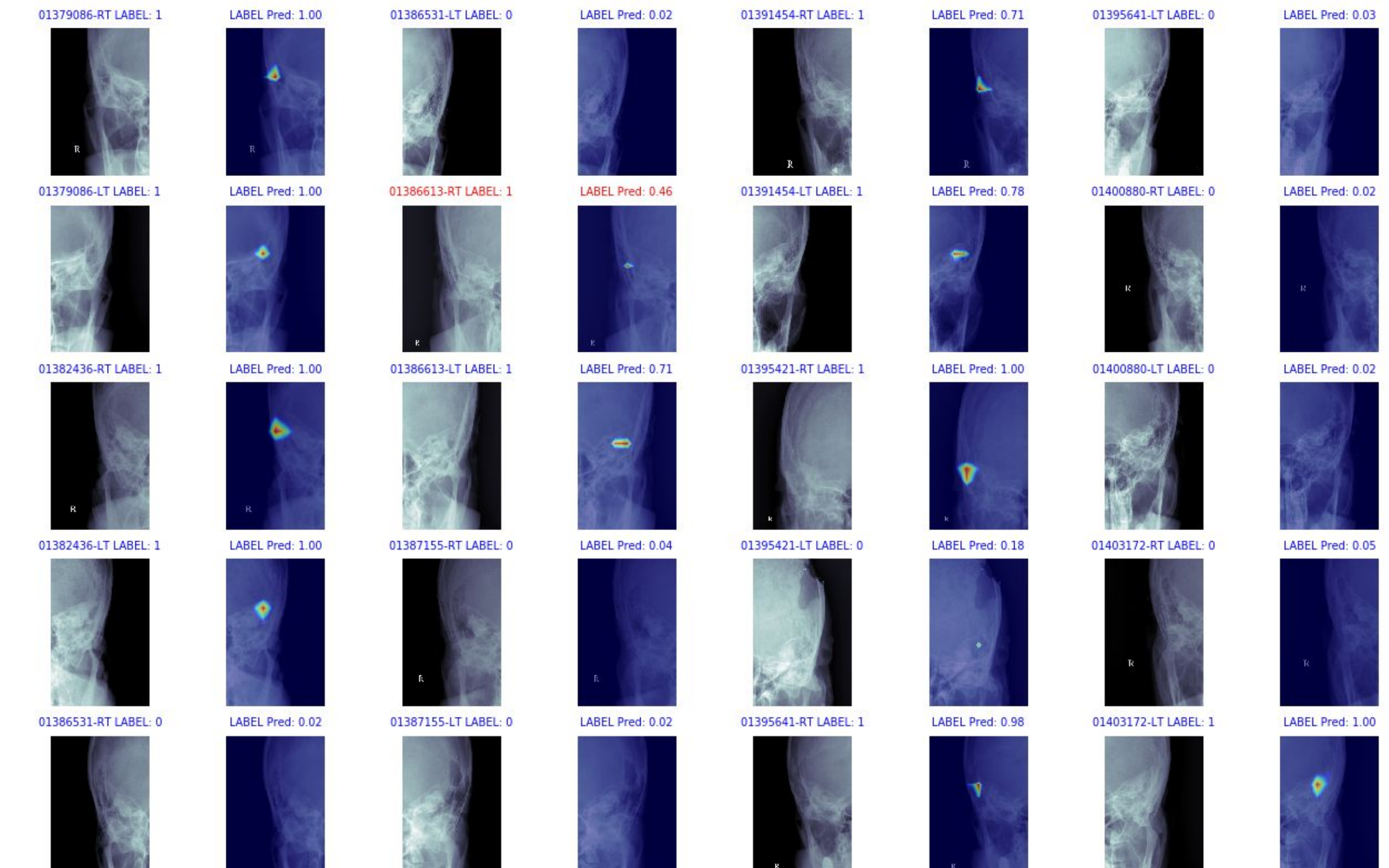

## Slide 33
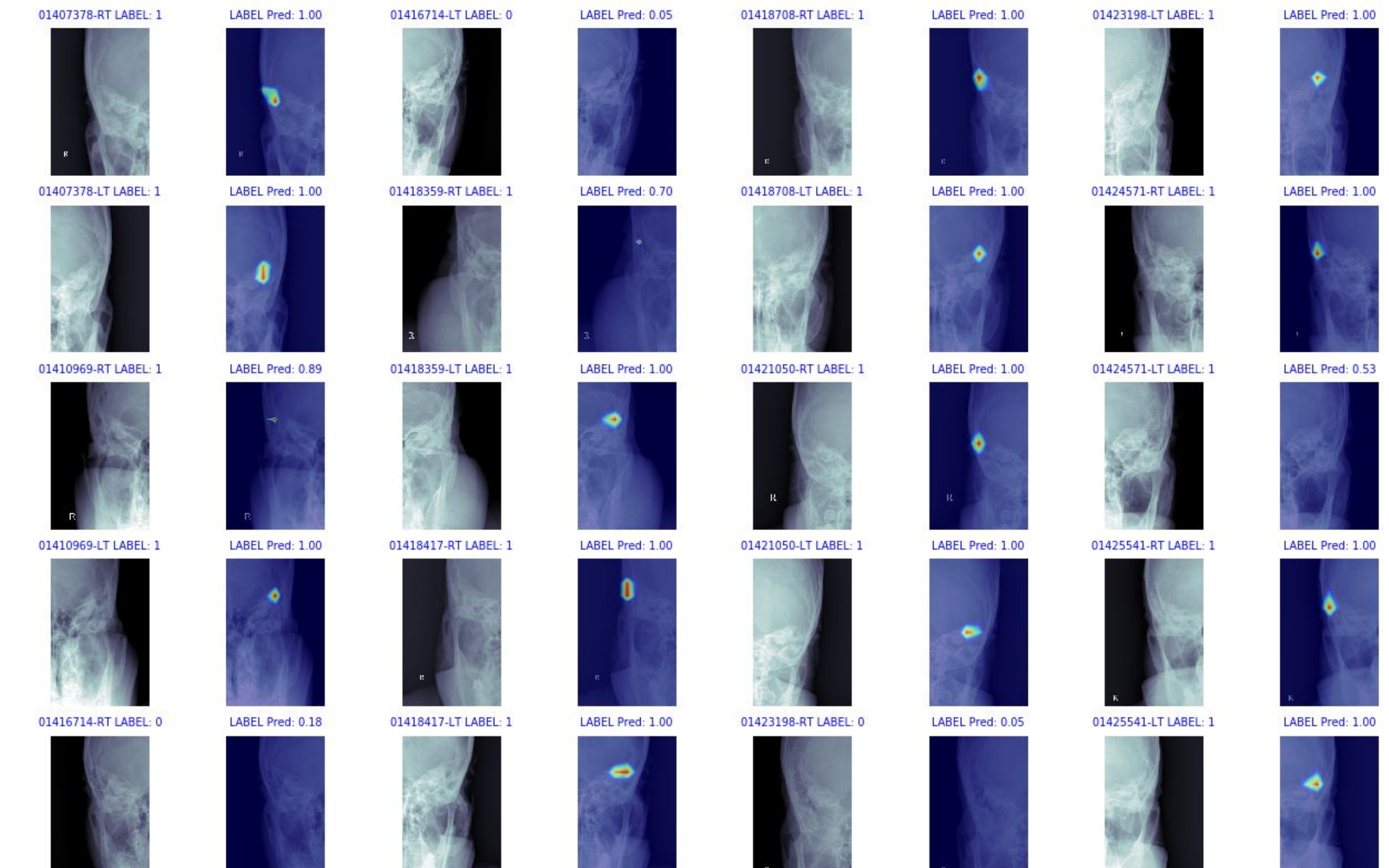

## Slide 34
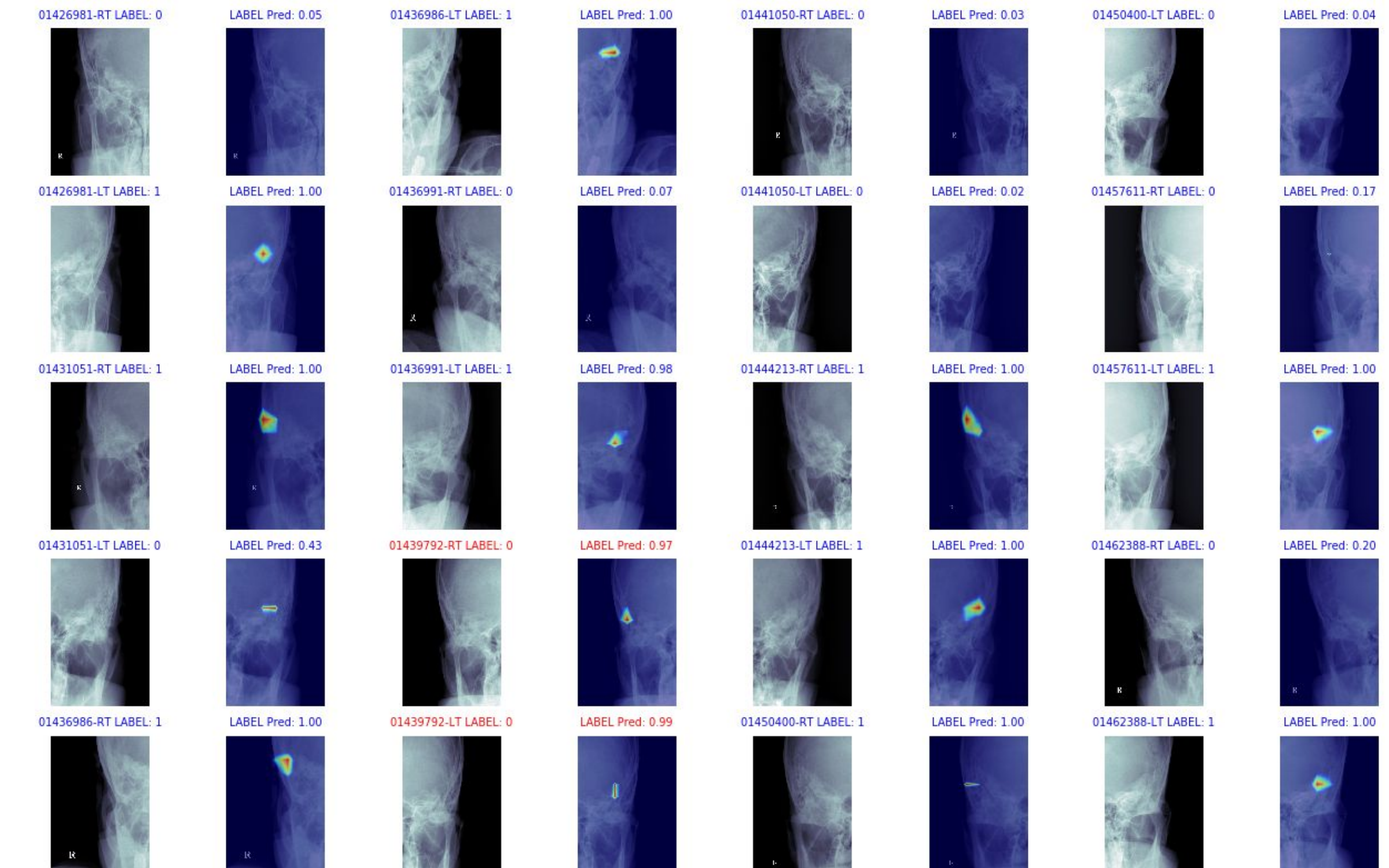

## Slide 35
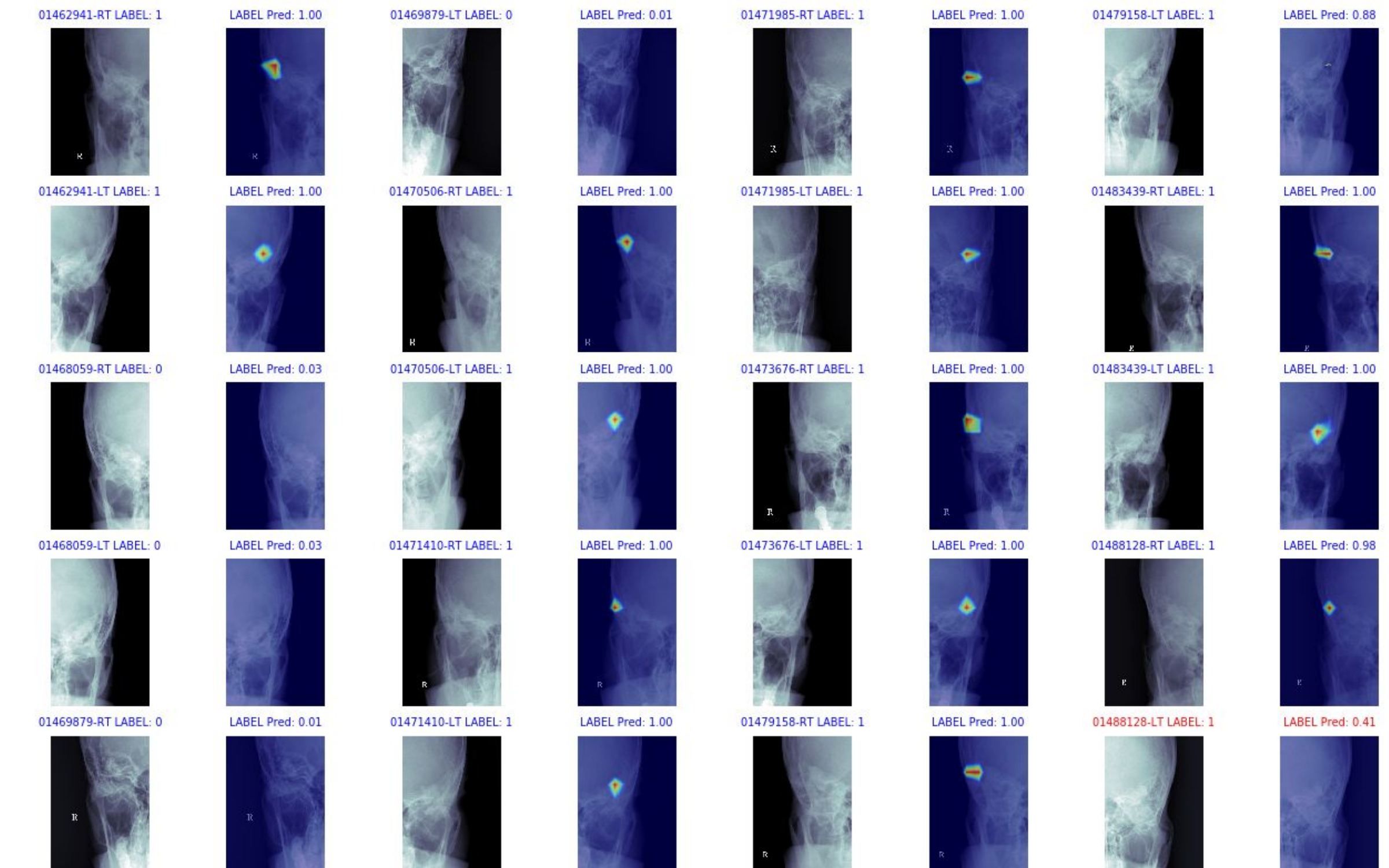

## Slide 36
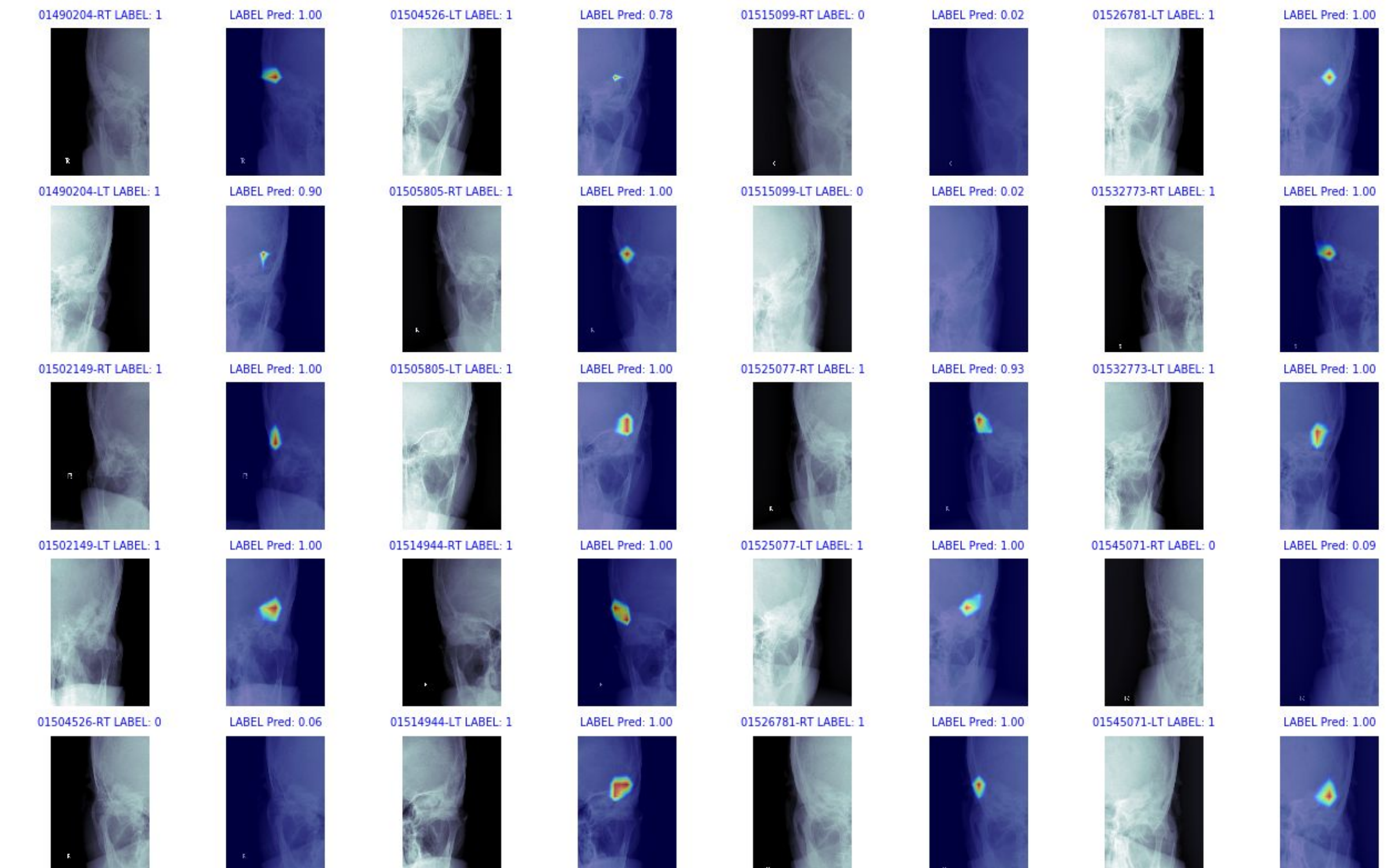

## Slide 37
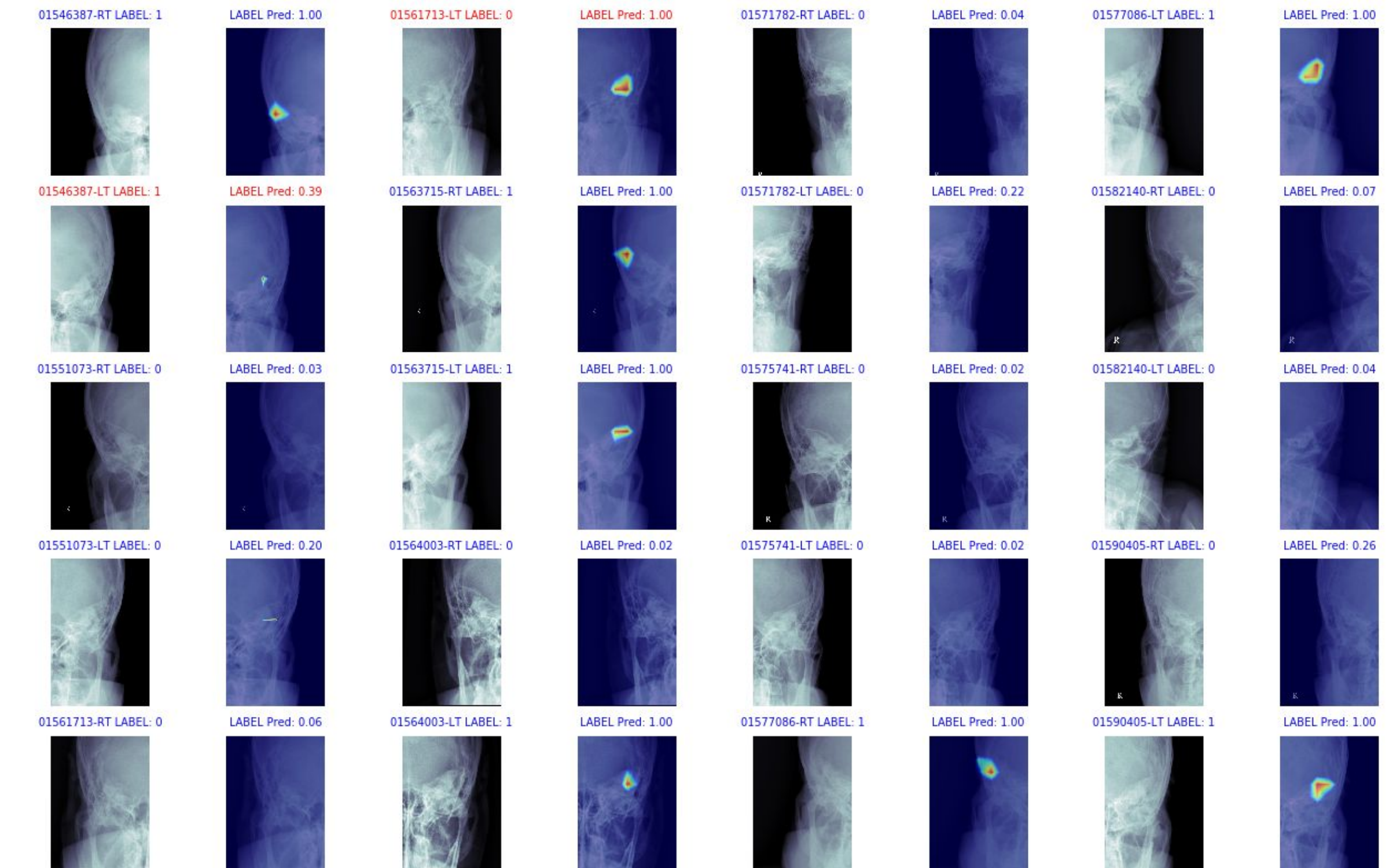

## Slide 38
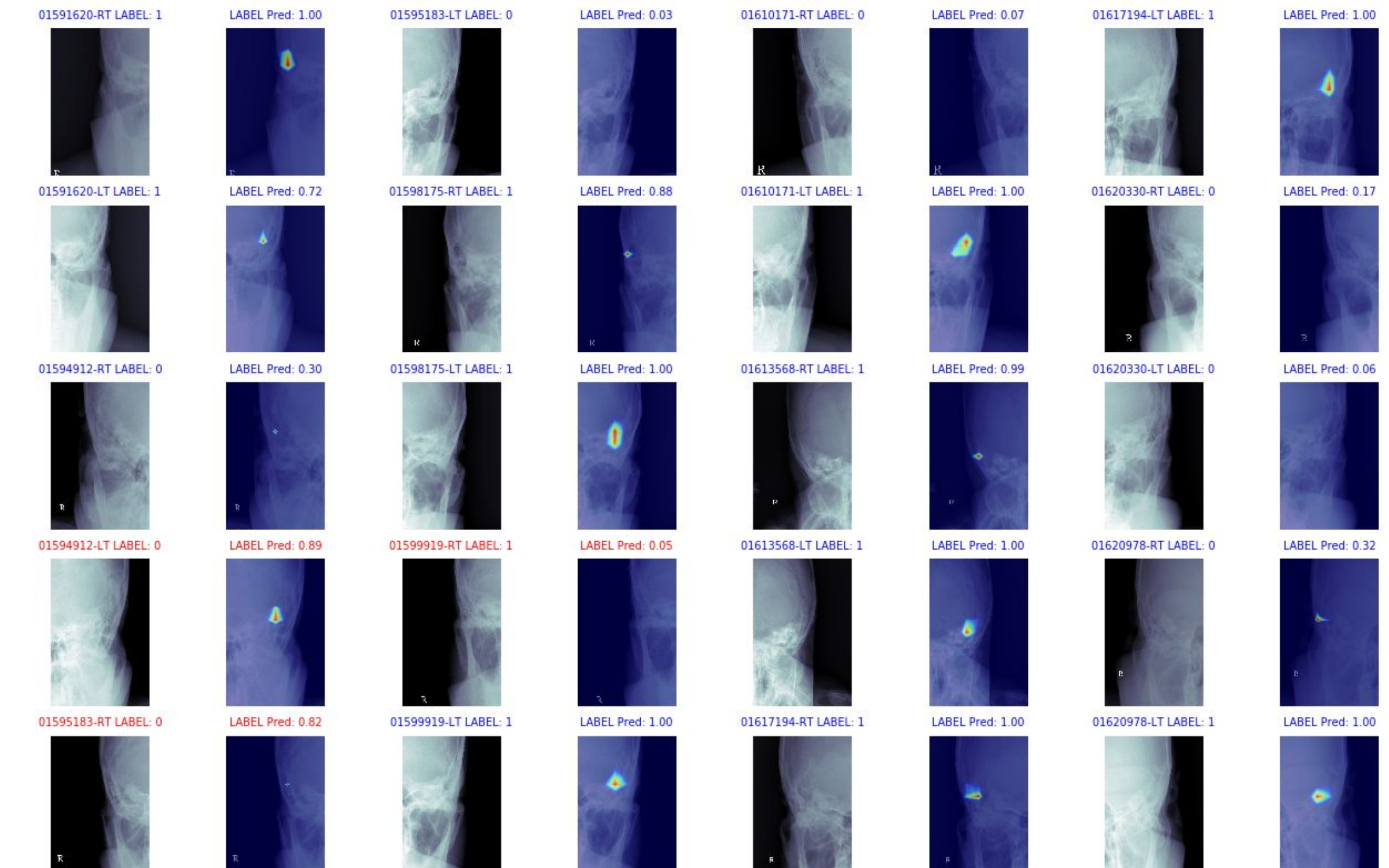

## Slide 39
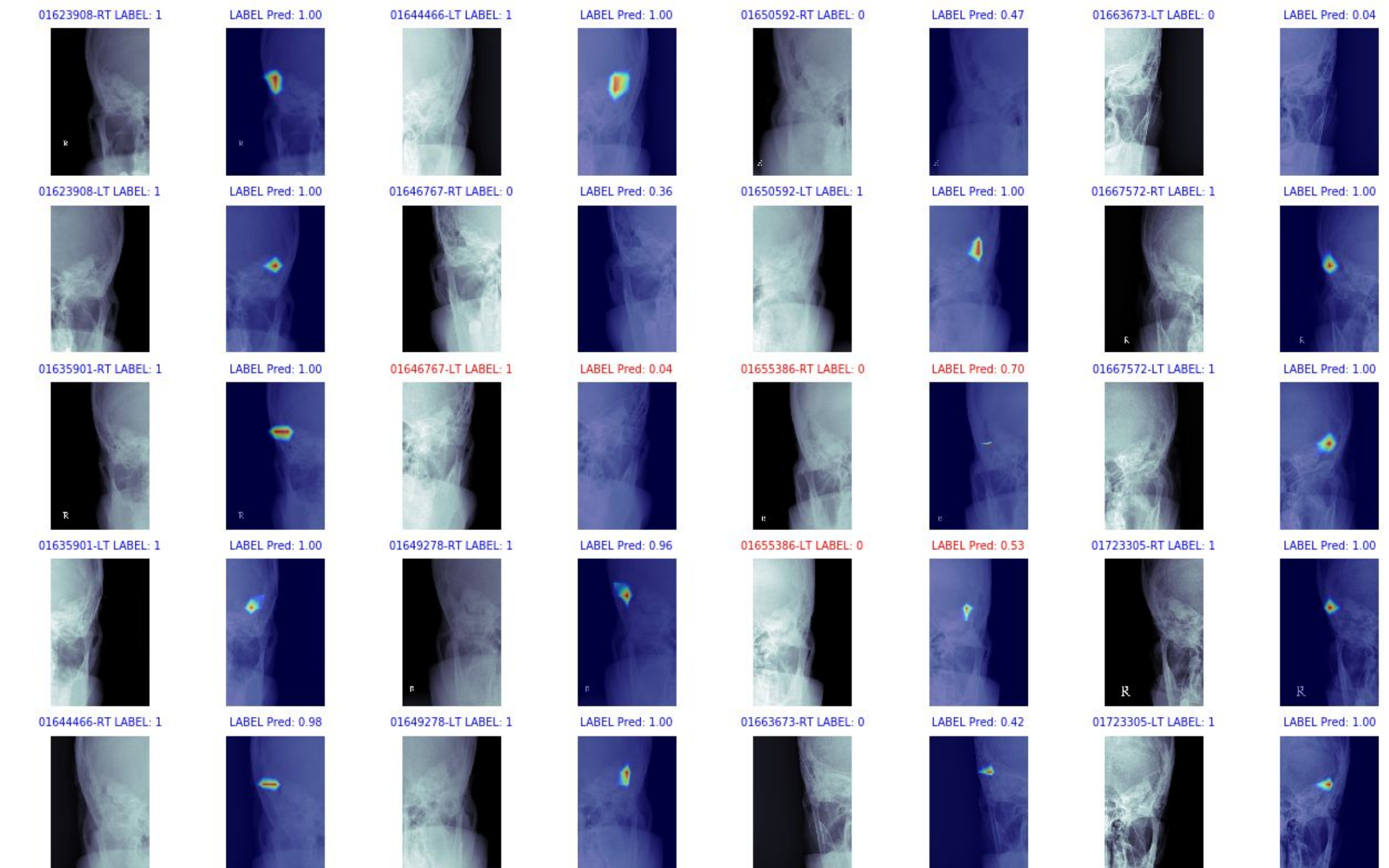

## Slide 40
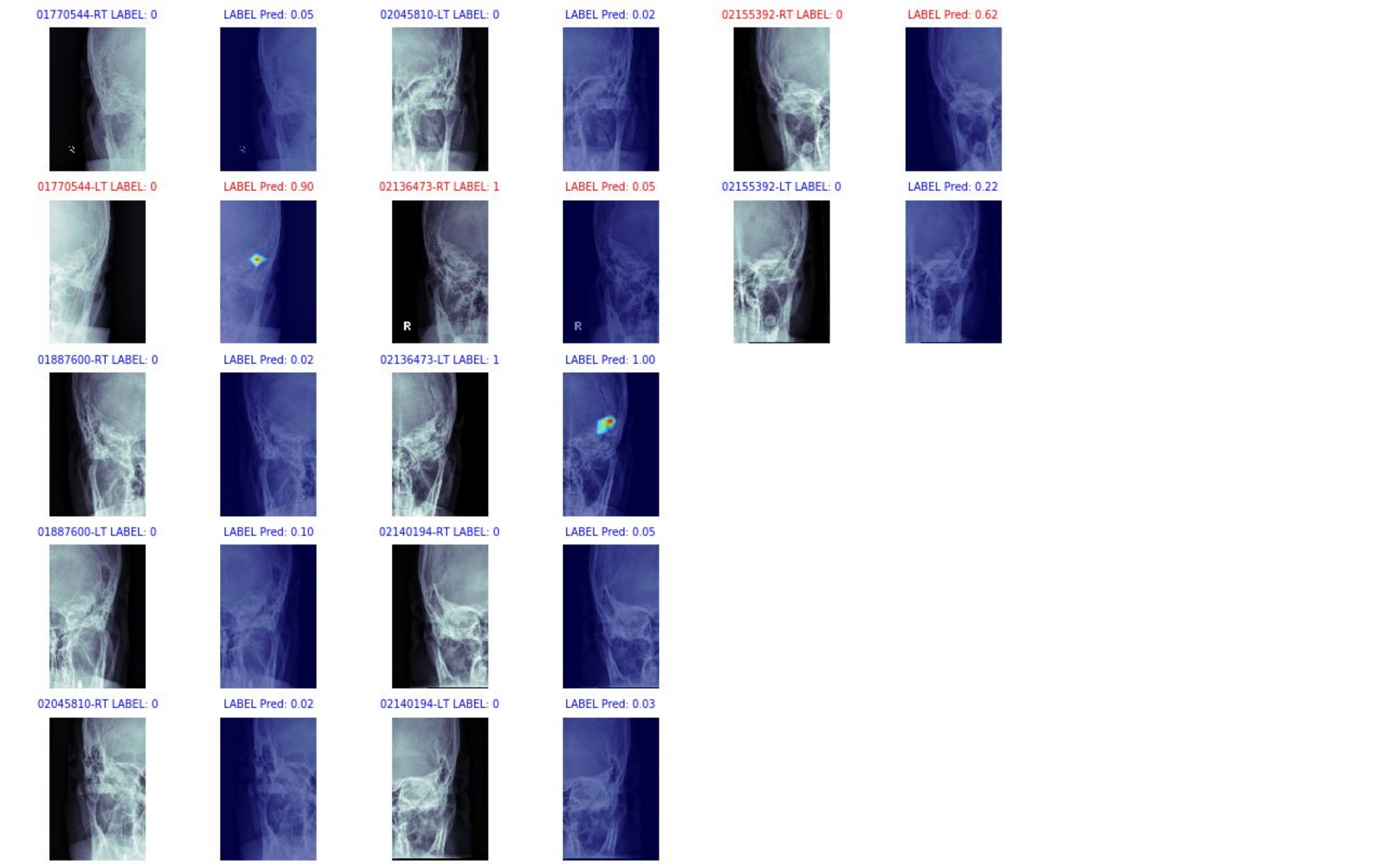

Supplement: S1 File — (ZIP) [file pone.0241796.s001.zip › all labels and activation map1/Model60_exp030_gdstd_all.npy_e256_view0_001.pptx]
