# Supplementary material for: Performance of deep learning to detect mastoiditis using multiple conventional radiographs of mastoid
Source: PLoS One. 2020 Nov 11;15(11):e0241796. doi: 10.1371/journal.pone.0241796 (PMC7657495; doi:10.1371/journal.pone.0241796)

## Slide 1
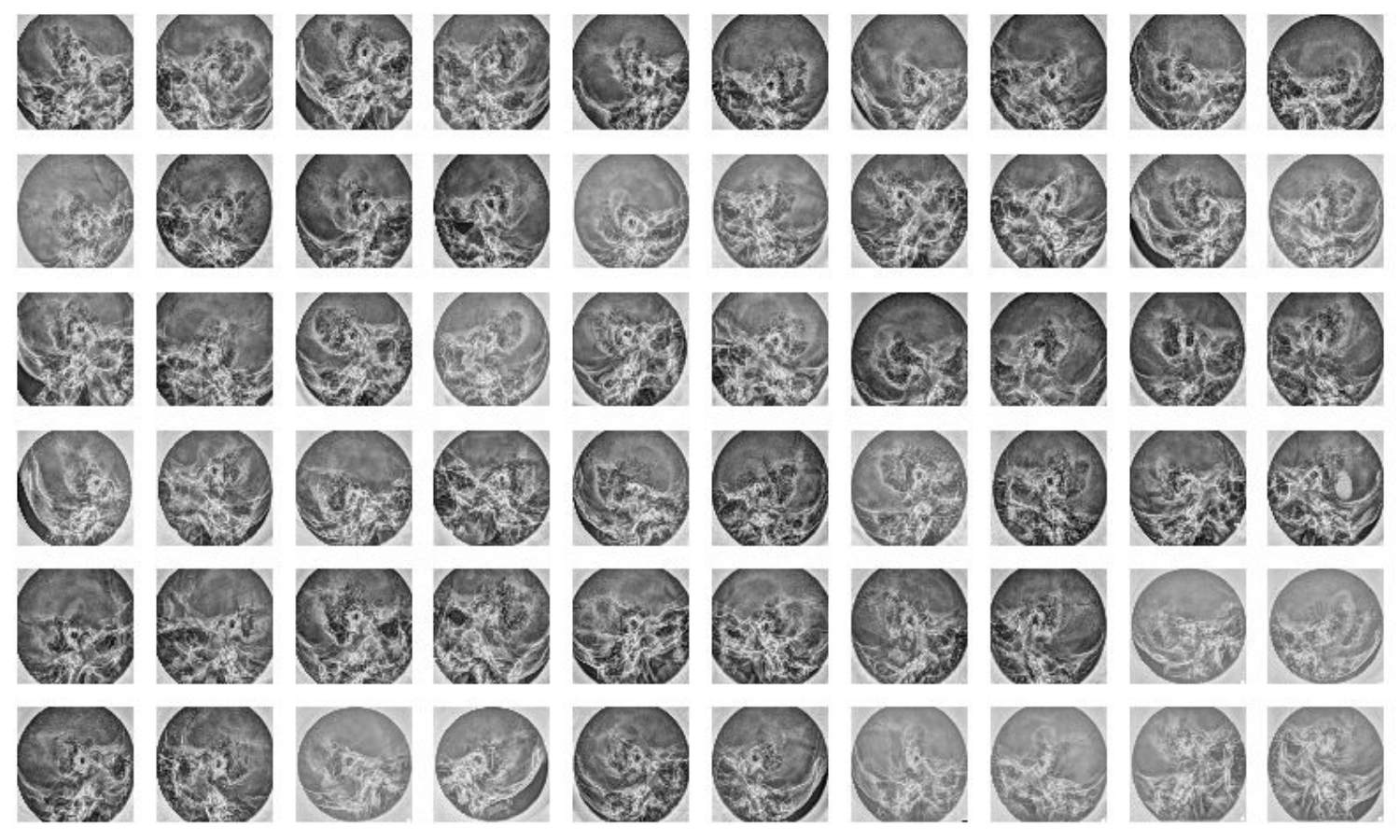

## Slide 2
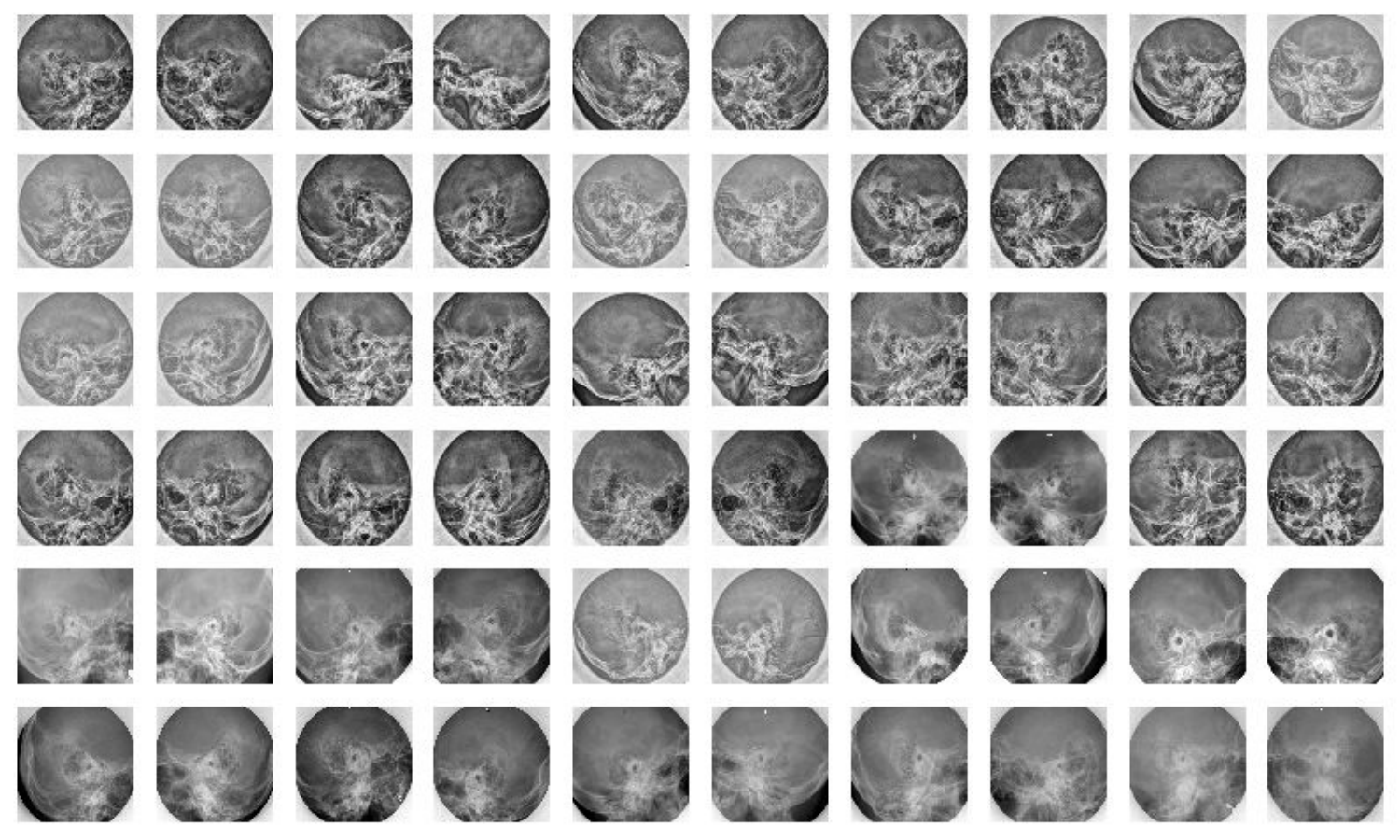

## Slide 3
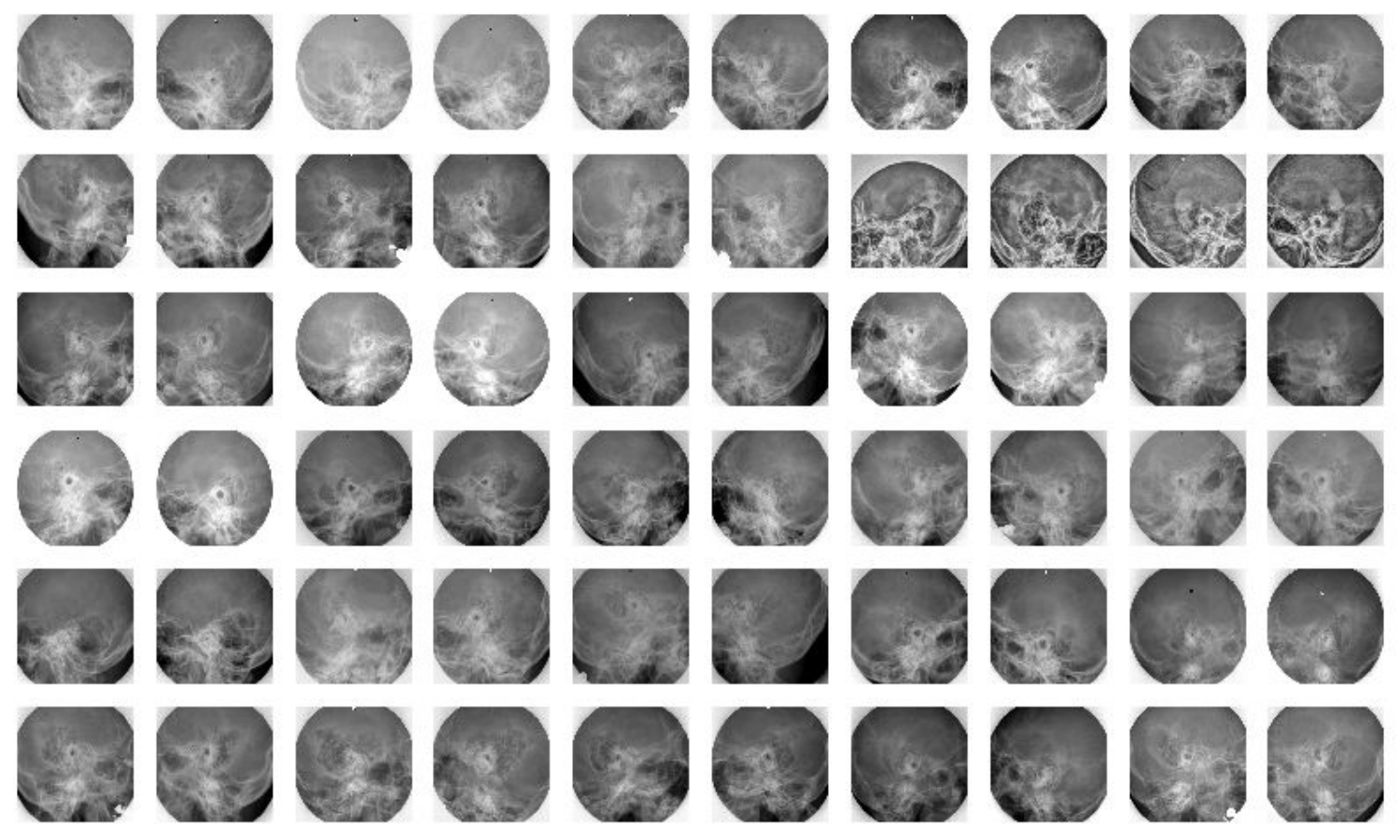

## Slide 4
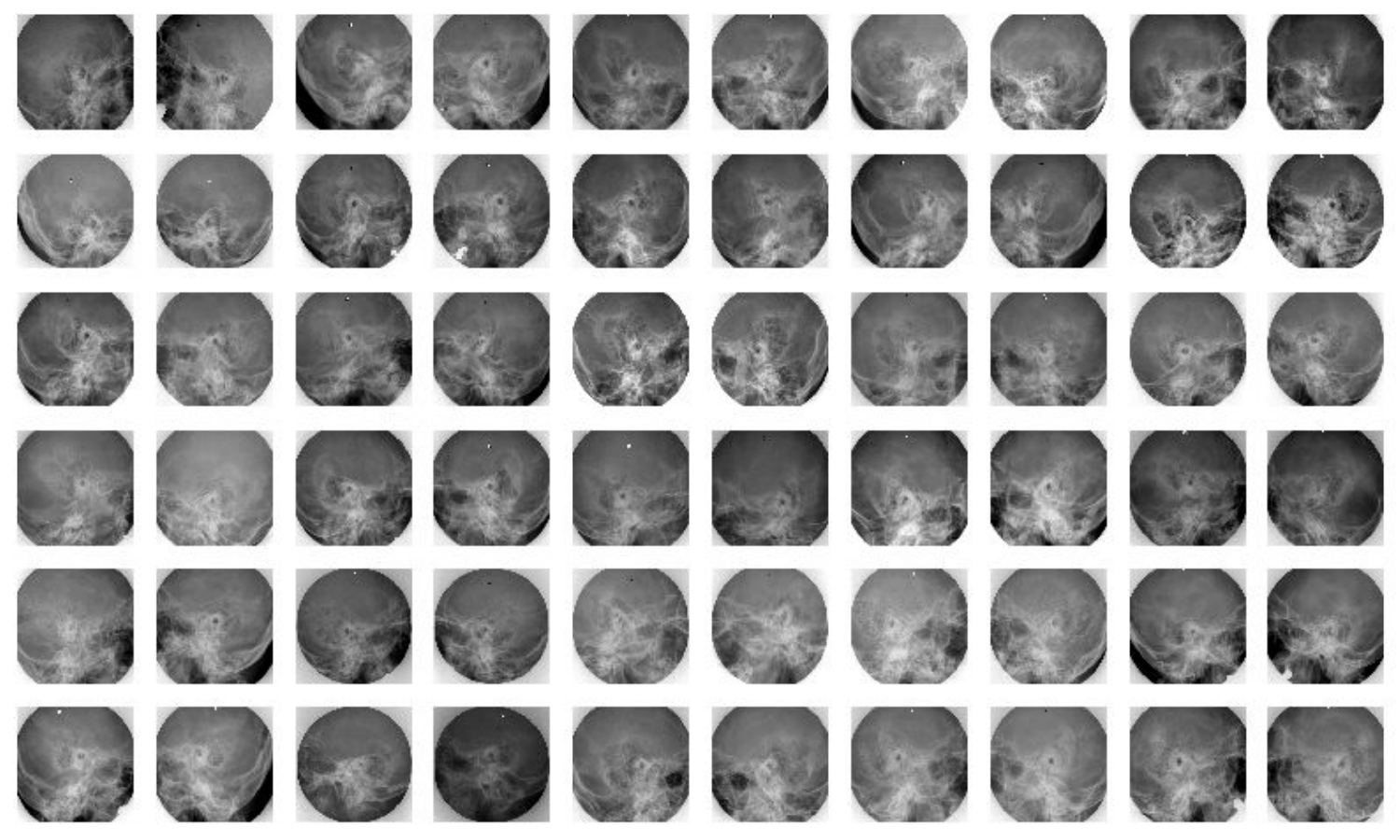

## Slide 5
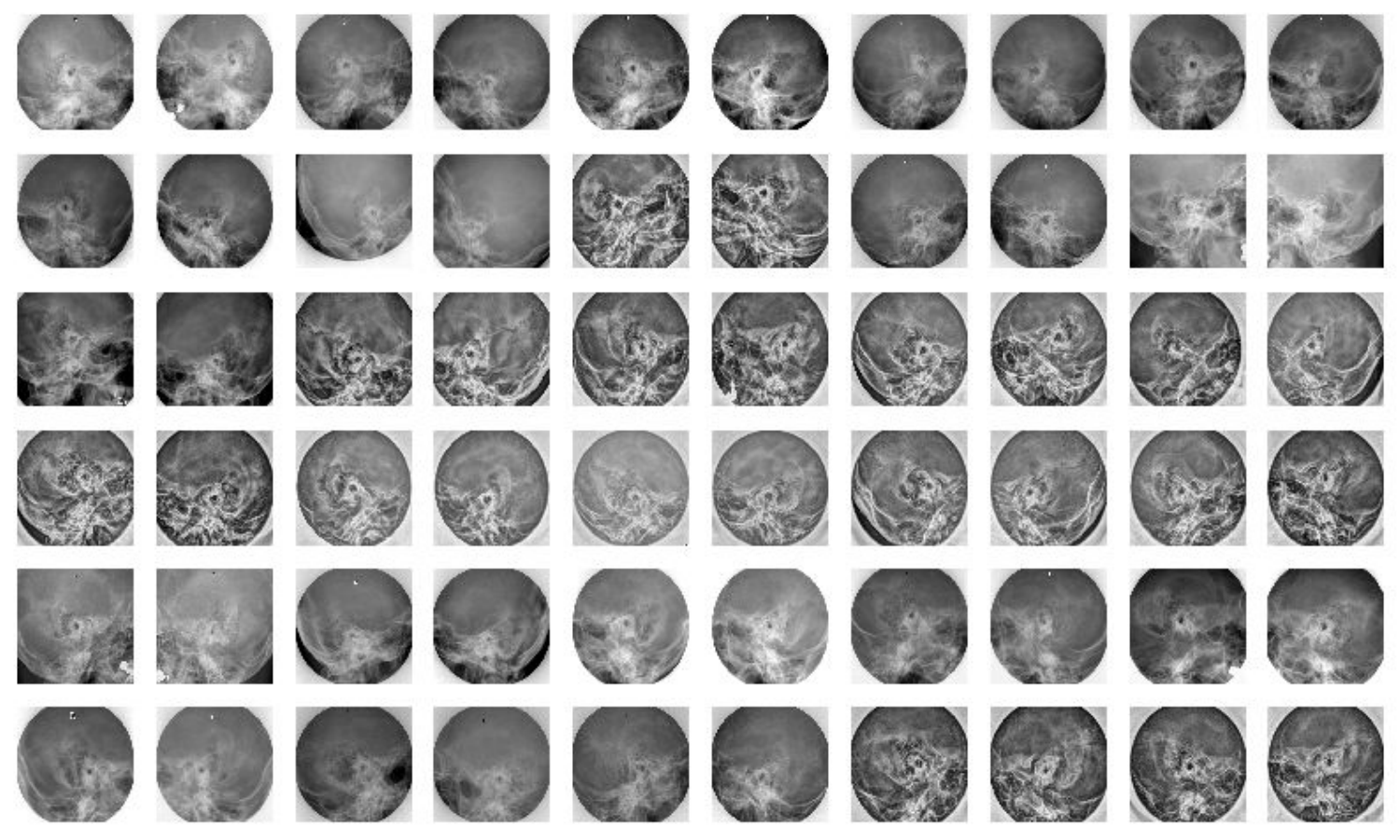

## Slide 6
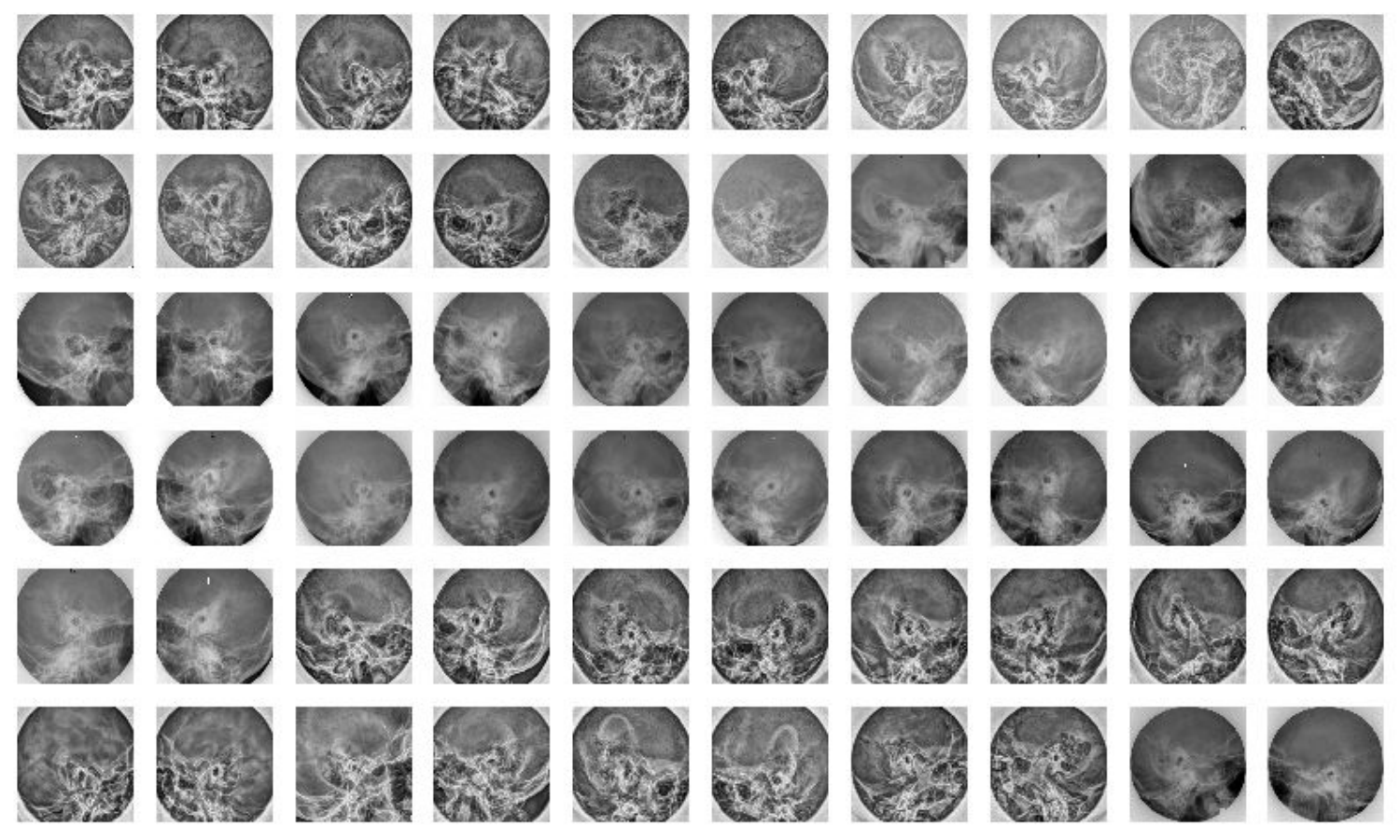

## Slide 7
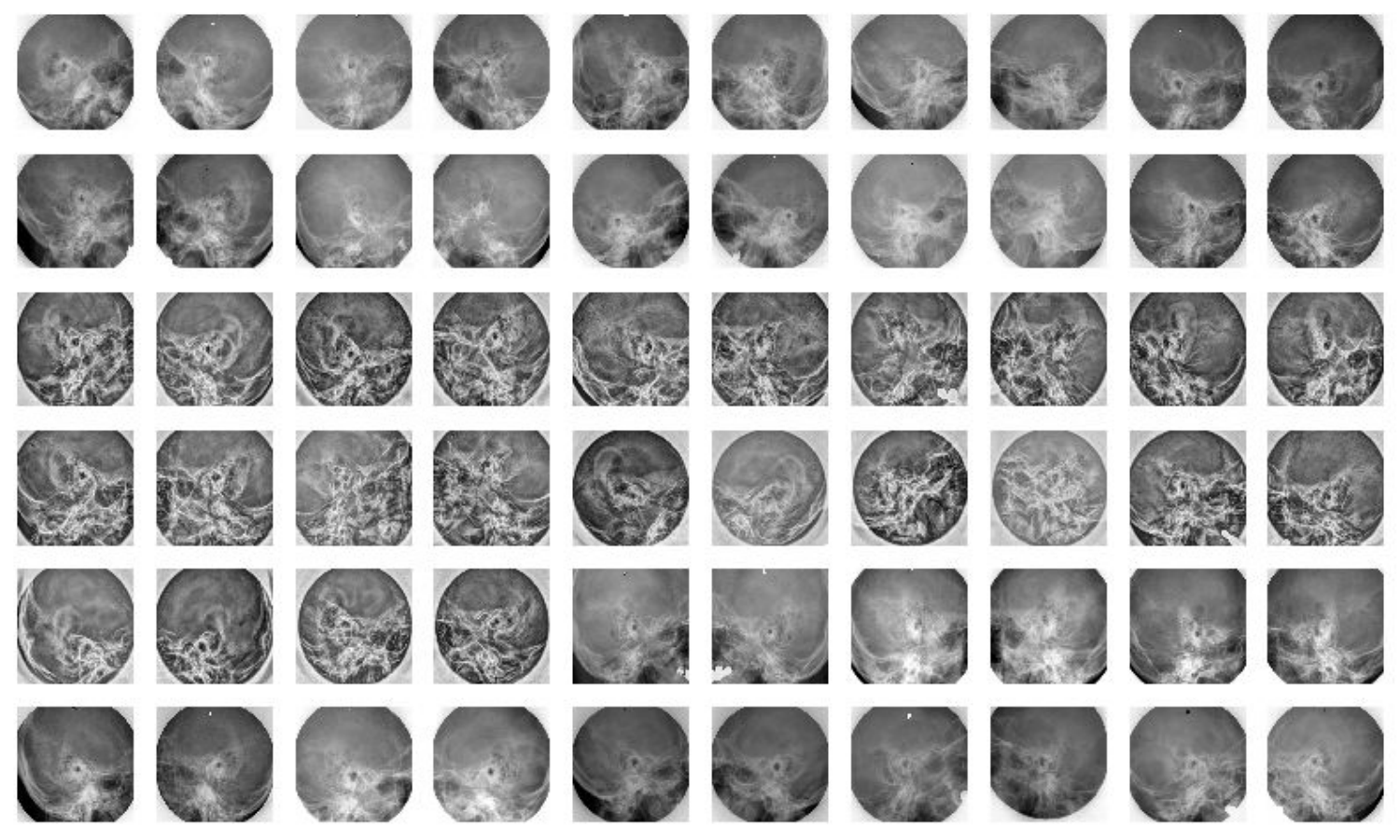

## Slide 8
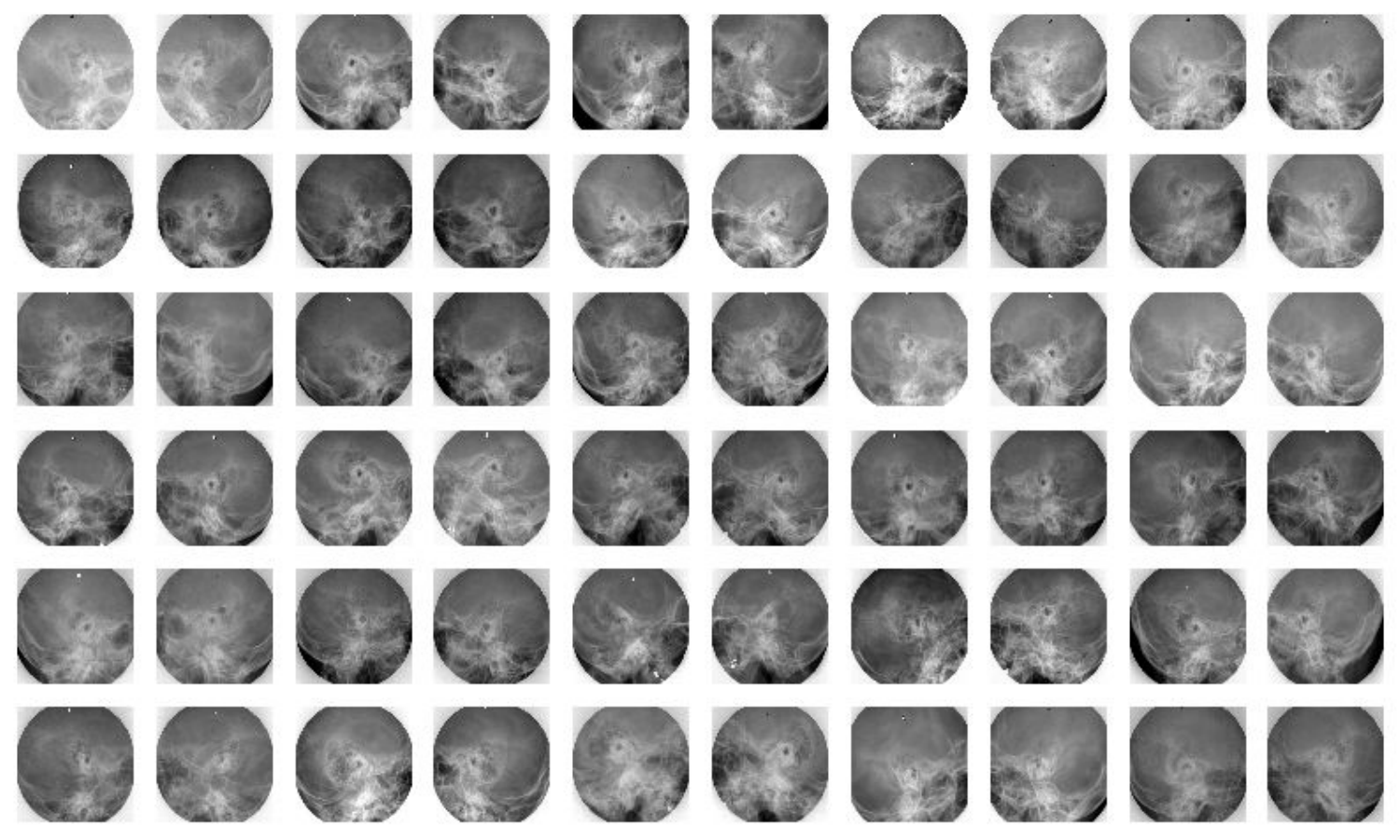

## Slide 9
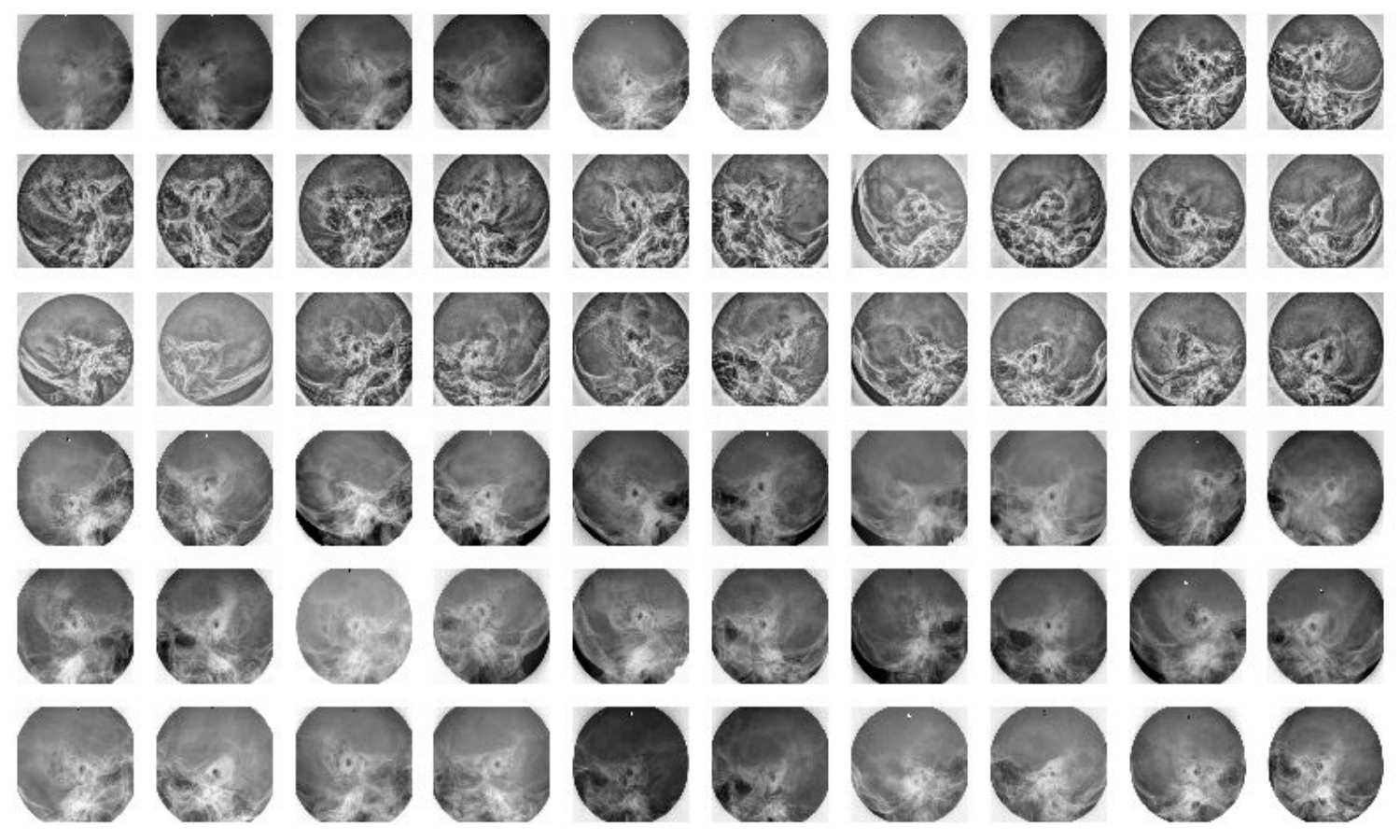

## Slide 10
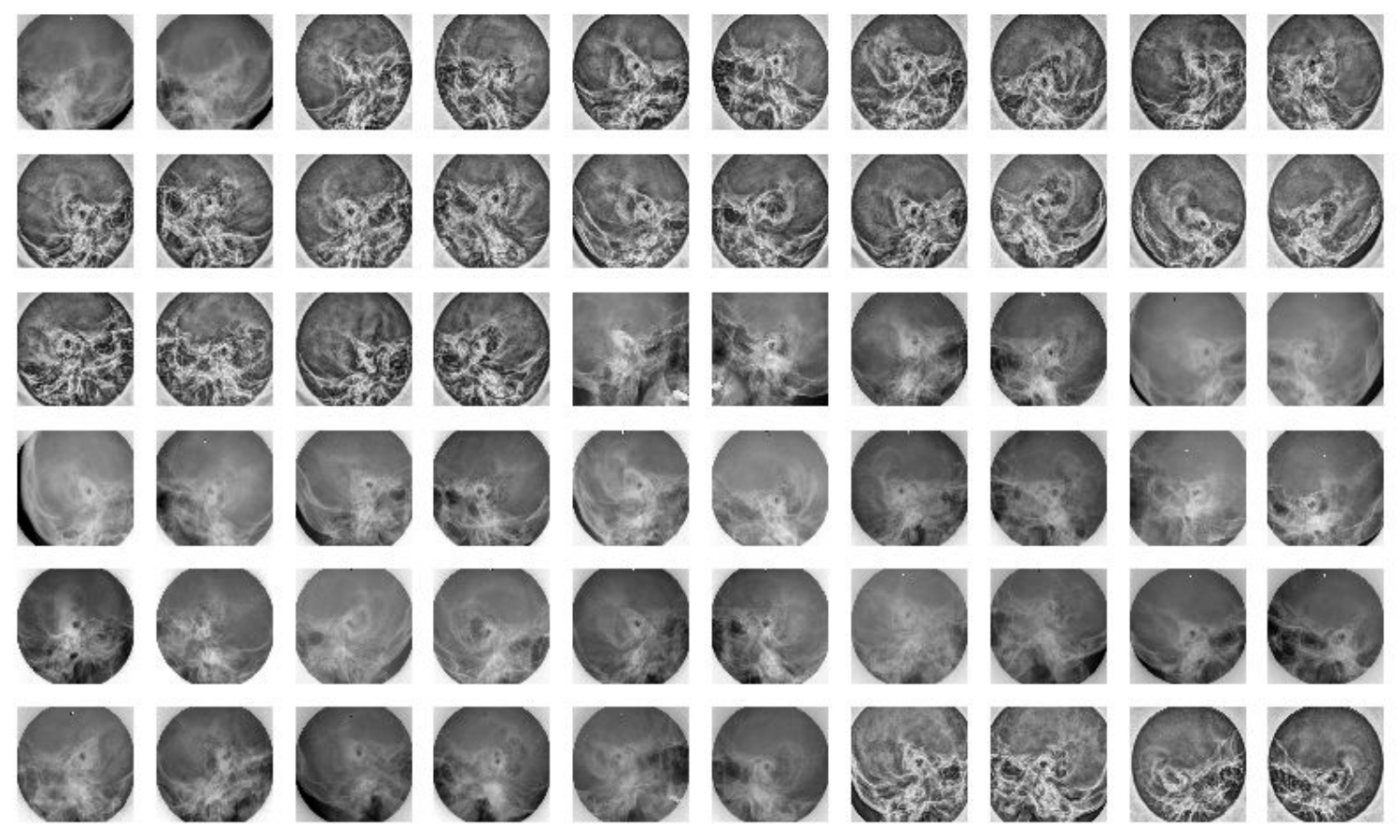

## Slide 11
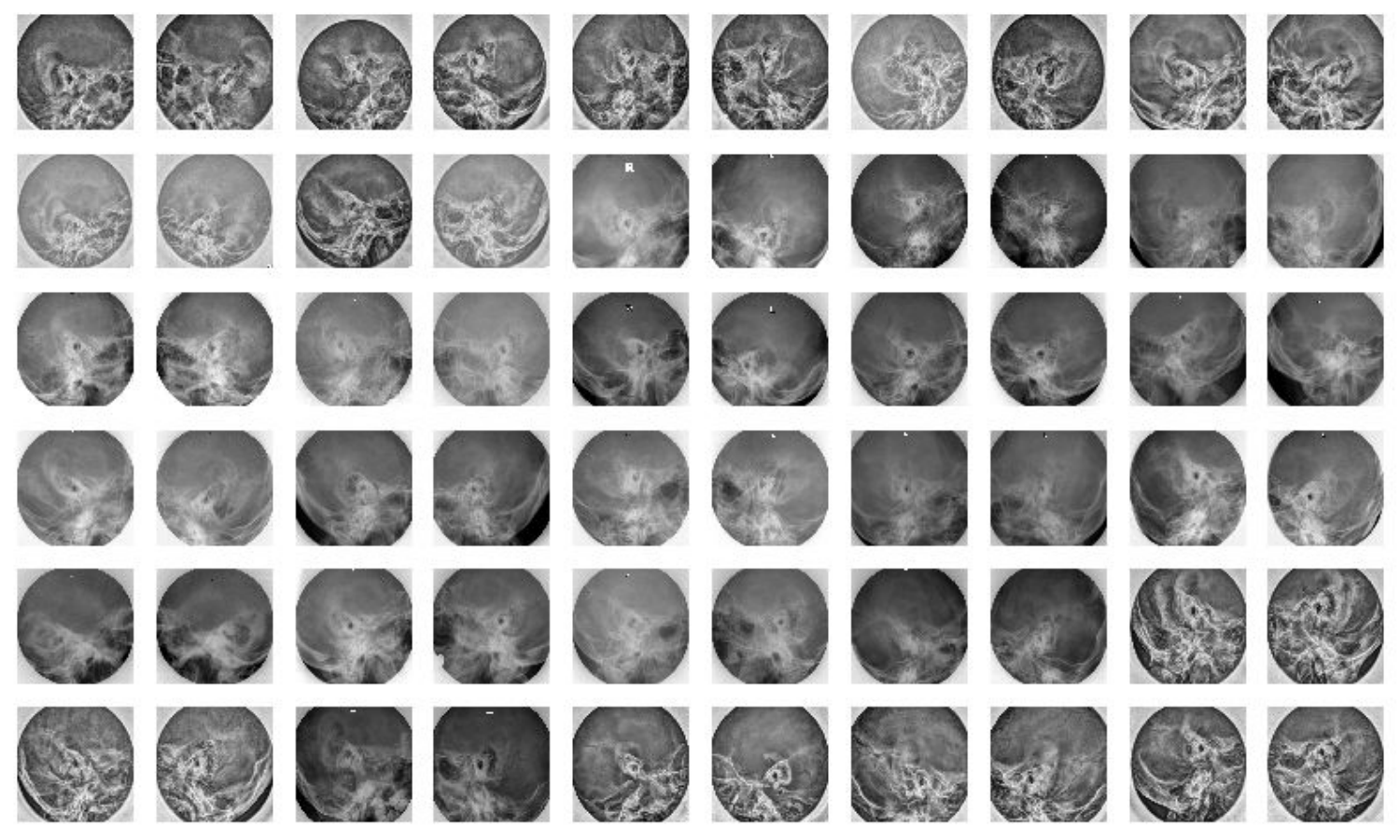

## Slide 12
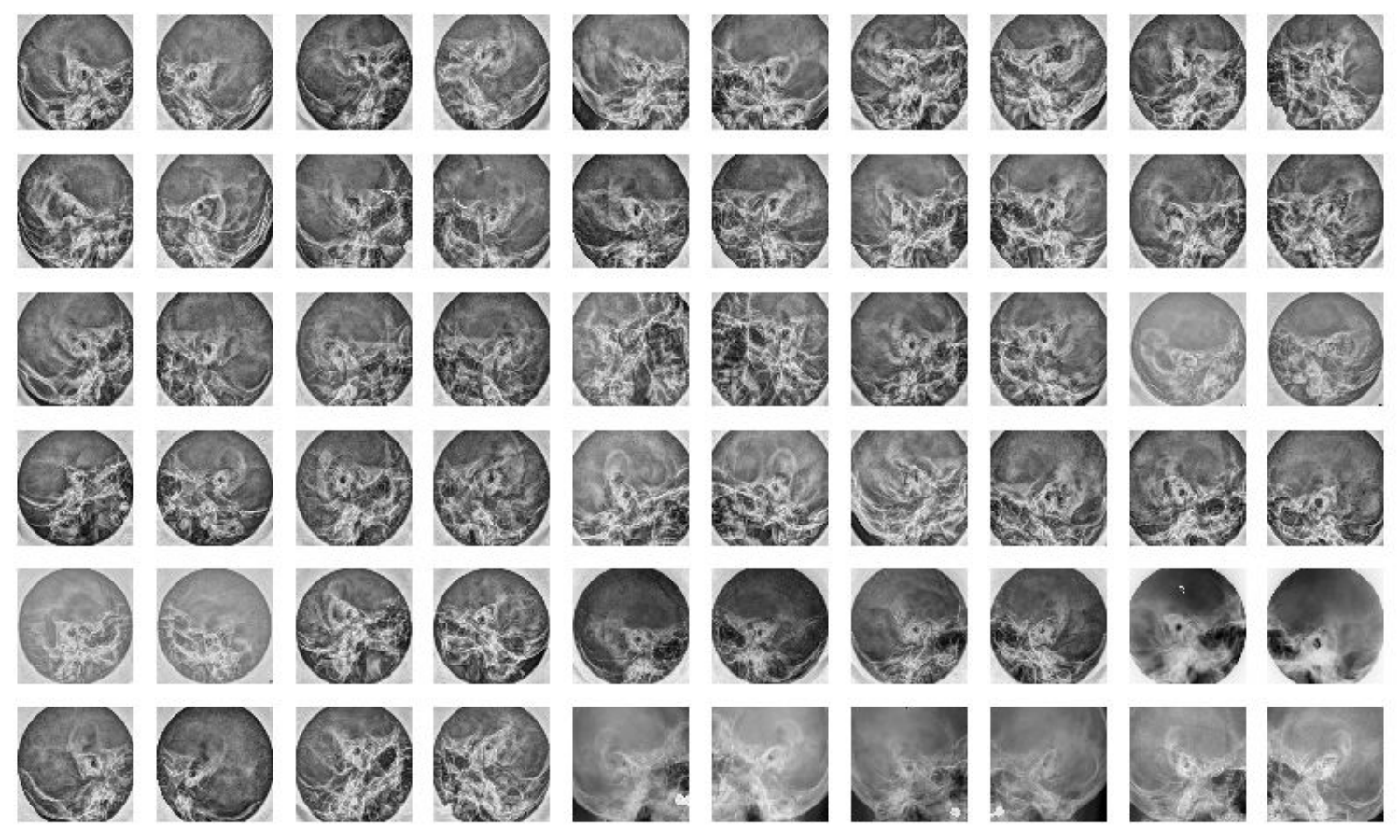

## Slide 13
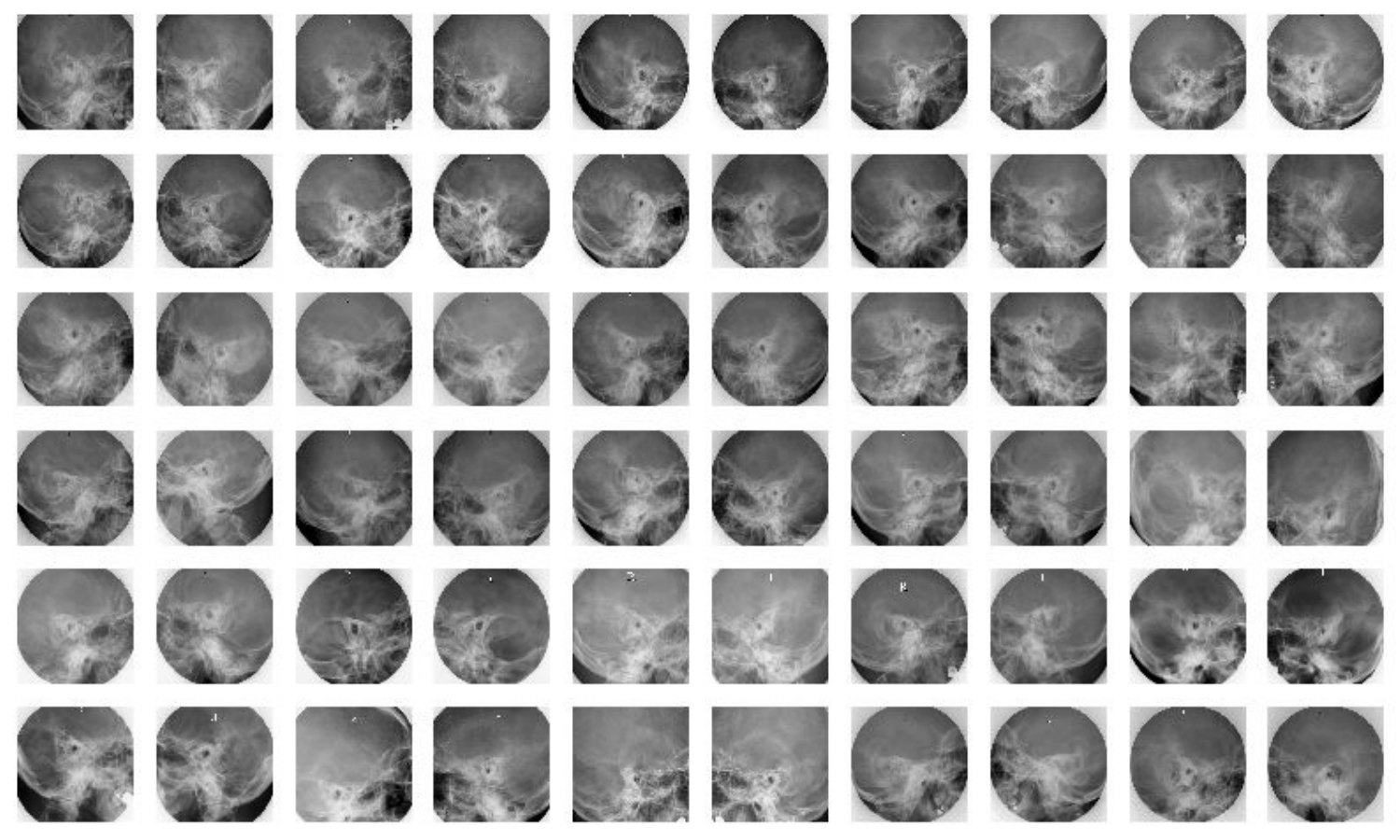

## Slide 14
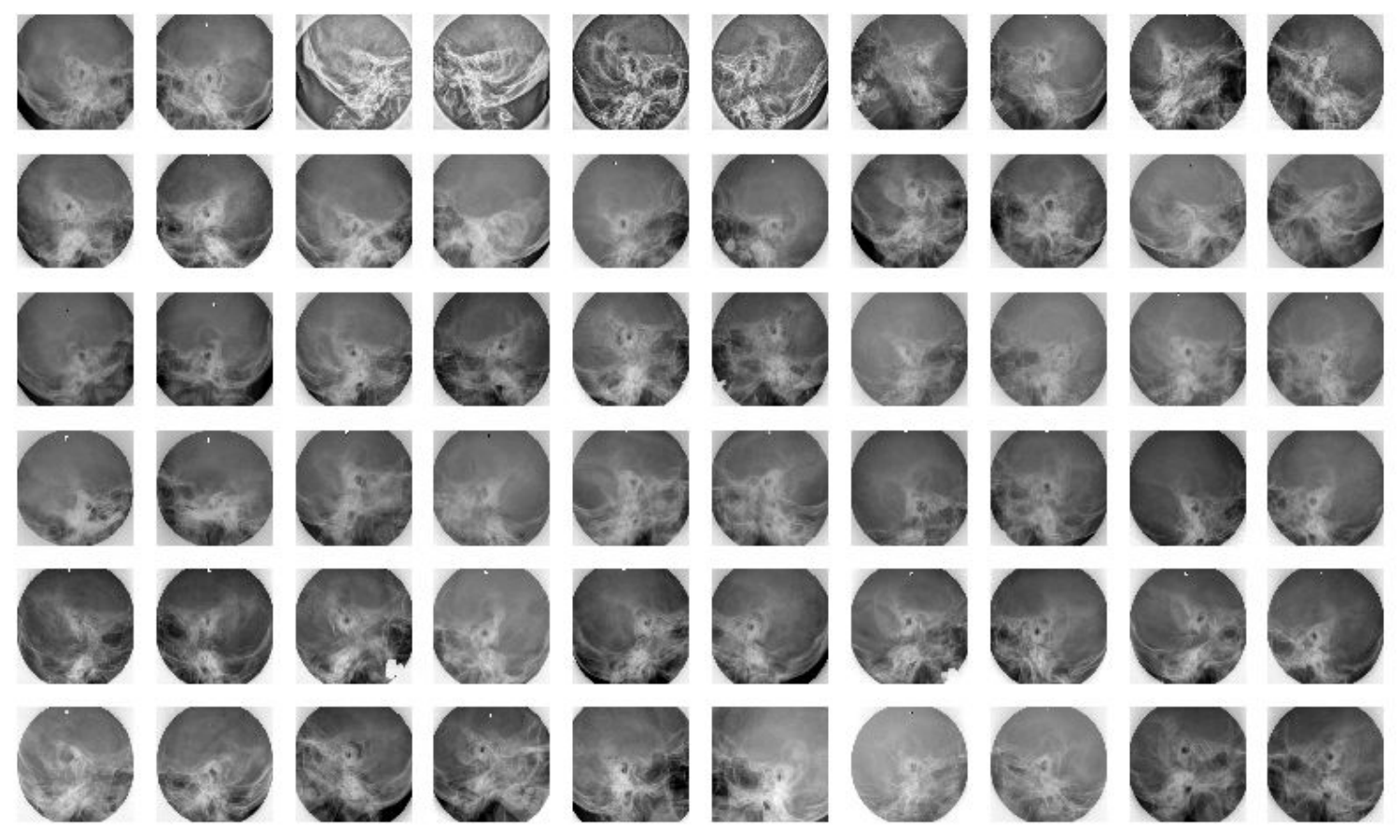

## Slide 15
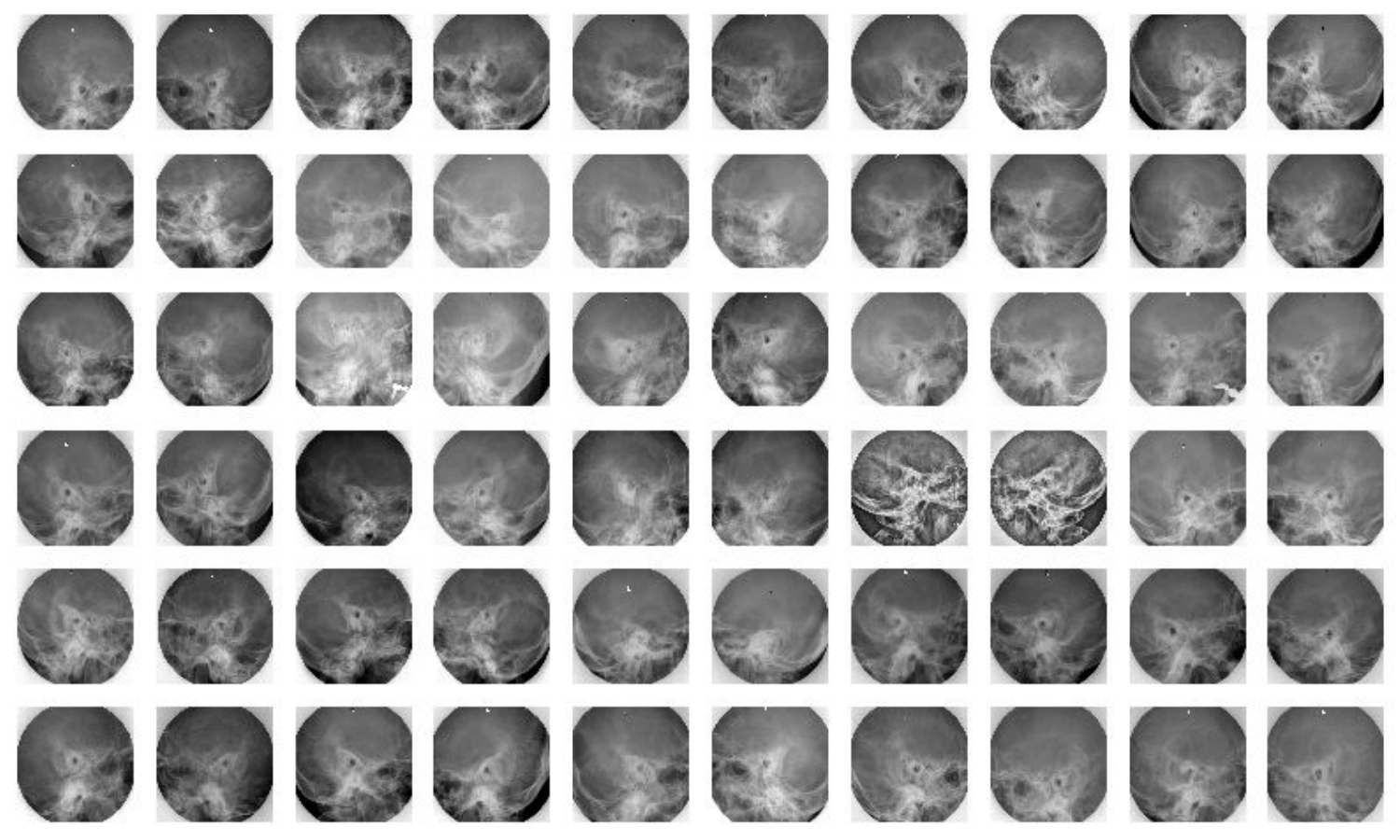

## Slide 16
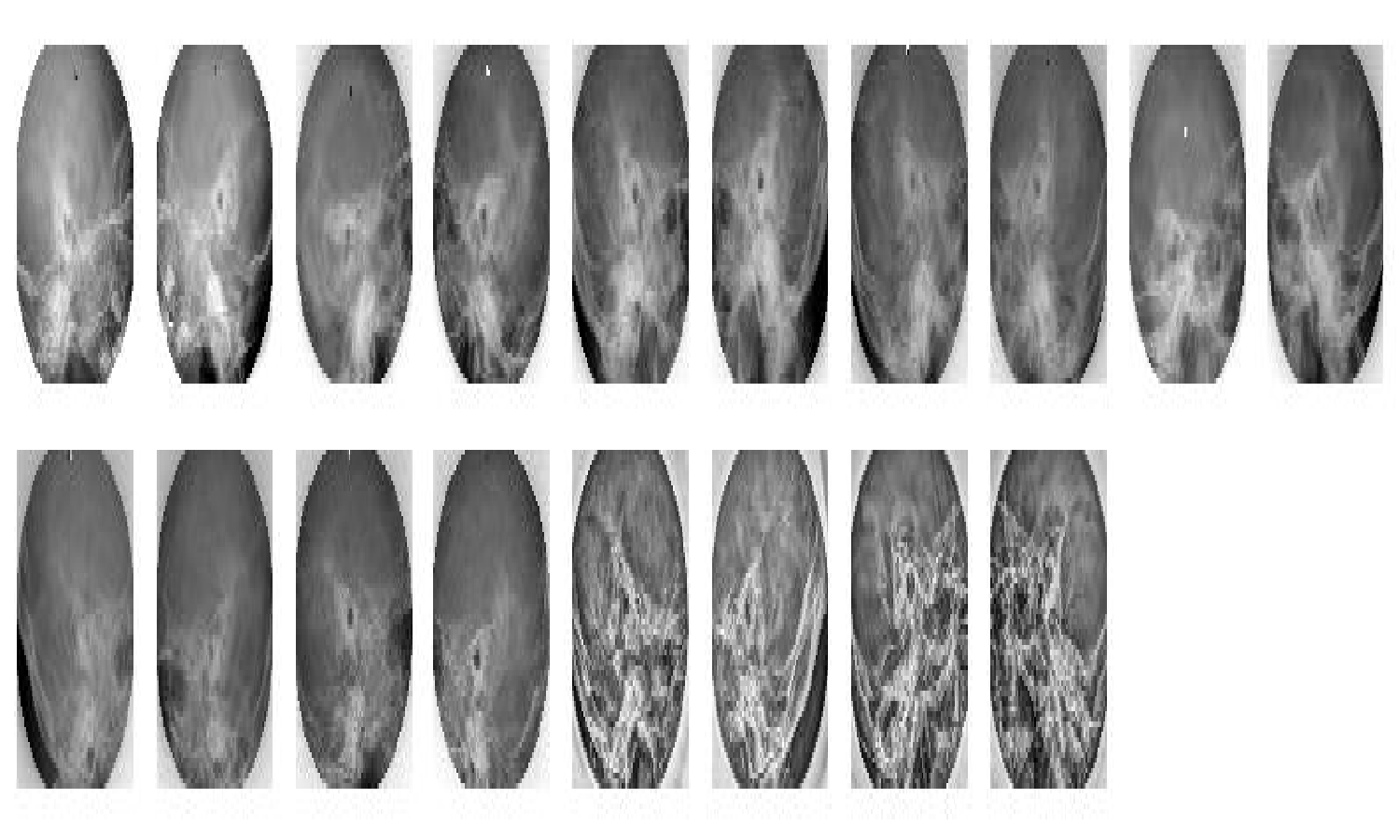

Supplement: S3 File — (ZIP) [file pone.0241796.s003.zip › all labels and activation map3/Validation_image_Lateral.pptx]
