# Supplementary material for: Performance of deep learning to detect mastoiditis using multiple conventional radiographs of mastoid
Source: PLoS One. 2020 Nov 11;15(11):e0241796. doi: 10.1371/journal.pone.0241796 (PMC7657495; doi:10.1371/journal.pone.0241796)
Supplement: Attachment — Submitted filename: response to the reviewer 20201005.docx [file pone.0241796.s005.docx]

Reviewer #1: The author partially address my concerns. The revision on the main text should be as important as comment response.

R1-1:The authors claim: “because the most commonly affected age group is the pediatric group, especially patients under two years old who are very sensitive to radiation exposure, simple radiography still has its role.”
But the age distribution in table 1 is not the case. 50-59 year-old patients contribute the most. Less than 20 year-old patients is minor, only around 8%. This compromised the novelty. Please explain it.

🡪 Thank you for your keen comment. It is known that the pediatric group can be more commonly affected by otomastoiditis than the older age groups (Ref. 2&6). However, the “affected” age here does not necessarily mean the most prevalent age group in radiologic examinations. Relative data shortage in this group may arise from other reasons. Unlike adult patients, many clinicians usually treat young patients without radiologic examinations in the first place. This is probably because young patients are more sensitive to radiation exposure than adults (Even though the radiation exposure of simple radiographs is much less than that of CT examinations) and/or performing examinations for children often involves various technical difficulties. Also, because organ transplantation surgeries are usually performed to adult patients, preoperative work up is much less frequent in children.

Even though our network was trained with the data set containing less exams of young patients, the evaluation result on the age group under 20 (accuracy=0.888, AUC=0.918) shows statistically non-inferior performance (p=0.928, p=0.125) compared with the other age group (accuracy=0.864, AUC=0.958); thus, the most affected group to the otomastoiditis can sufficiently benefit from the developed algorithm as well.

R1-2 “Deep learning algorithm depicted the exact location of diseased mastoid air cells.” The activation map of diseased mastoid air cells is a key contribution of this article. However, this article lacks of the detail implement of activation map in the method. Please add it.

🡪We appreciate your comment. The process of obtaining the class activation mapping in the deep learning algorithm part was described in more detail. In the manuscript, the class activation mapping obtained from CNN for multiple views was presented, and related parts were added. (L187-195, L322) In addition, the code we implemented was uploaded to the public repository and the related part was added. (L184-185)

R1-3 Please provide a reference for the claim of “Simple radiographies take a large portion of radiologists’ work-loads.” (L47-48)

🡪As we mentioned in the manuscript, simple radiographs are the most commonly performed imaging procedures in the US and the average radiologist reads more than 100 chest radiographs per day. (Ref. 7,8) However those references focused on chest radiographies (of course, the chest radiographs take major part of all radiographs). So we added another reference (Ref 9: Trends in Diagnostic Imaging Utilization among Medicare and Commercially Insured Adults from 2003 through 2016, Radiology 2020:294:342-350) which showed that more than a half of the diagnostic imaging performed from 2003 through 2016 in the US are simple radiographies. In this article, the diagnostic imaging modalities included even non-radiologists’ works (e.g., echocardiography, Nuclear imaging). Nonetheless, more than a half were simple radiographies. (L48)

R1-4:Line 66 What is the inclusion criteria in detail? Do somehow mastoiditis need a screening? Why the mastoid series didn't include any other diseases?
--> Mastoid series were usually performed for screening mastoiditis (infection/inflammation) before operations such as organ transplantation and cochlear implantation. In those cases, preoperative treatment of mastoiditis is very important. (immunosuppresants will be used in patients after organ transplantation surgery, cochlear implant electrodes will go through mastoid air cells in cochlear implant op) Also, mastoid series were also performed in patients with suspected mastoiditis. (L69)
Mastoid series were used for detecting mastoiditis. Other diseases are very rare in mastoid air cells and also even those rare diseases (such as tumorous condition) are usually presented as mastoiditis patterns in imaging.
Please add this background information to the paper.

🡪 Thank you for your kind comment. We added this content in the manuscript. However, some of the contents are too long and a little bit out of focus in M&M section (e.g., “other diseases such as primary tumors are very rare in the mastoid area and even those diseases are presented as mastoiditis” is a common sense to doctors), we added some parts of these contents. (L73-76)

R1-5 Actually majority of cases in gold standard test sets (which have both mastoid series and TB CT) performed TB CT and mastoid series simultaneously. 
Please add this response to the paper.
🡪 According to your suggestion, we added this information in the manuscript. (L81-82)

R1-6 L96 please revise to labels were determined by consensus after the two radiologists discussed.
🡪 We appreciate your kind suggestion. According to this suggestion, we revised the sentence. (L103-104)

R1-7 It seems I.R. read both CT and plain radiography. Yes. However, labeling TB CT and labeling radiographs were performed separately (blinded). When labeling the plain radiographs of gold standard set by I.R., those plain radiographs (around 800 image sets) were randomly mixed with other 9,000 images in training/validation sets.
Please add this response to the paper.

🡪 Thank you for the comments, we added this response in the manuscript. (L114-116)

R1-8 The left/right center points in the AP view are the points annotated in the image below. Outliers are excluded from the analysis.(L135)
Please add this response to the paper.
🡪 We added this content in the manuscript.(L147-149) The AP view image of the points annotated was also added (Fig 2).

R1-9:Line 126 Do horizontal and vertical shift on the original image or on the cropped image with zero paddings?
--> Horizontal and vertical shift were used in the training process.
The authors didn’t answer this question.
Image shift will result in black edge. Shifting on the original image before cropping will replace the black edge with the pixels adjacent to the edge.
Did you do it on the original image or on the cropped image?

🡪 We certainly did the shift operation on the “original” images and avoided the black edge problem. Thank you for reminding us.
